# An explainable artificial intelligence approach for decoding the enhancer histone modifications code and identification of novel enhancers in Drosophila

**DOI:** 10.1186/s13059-021-02532-7

**Published:** 2021-11-08

**Authors:** Jareth C. Wolfe, Liudmila A. Mikheeva, Hani Hagras, Nicolae Radu Zabet

**Affiliations:** 1 School of Life Sciences, University of Essex, Colchester, CO4 3SQ UK; 2 School of Computer Science and Electronic Engineering, University of Essex, Colchester, CO4 3SQ UK; 3 Blizard Institute, Barts and The London School of Medicine and Dentistry, Queen Mary University of London, E1 2AT, London, UK; 4 Department of Mathematical Sciences, University of Essex, Colchester, CO4 3SQ UK

**Keywords:** Enhancers, Histone modifications, Explainable Artificial Intelligence, Gene regulation, Drosophila

## Abstract

**Background:**

Enhancers are non-coding regions of the genome that control the activity of target genes. Recent efforts to identify active enhancers experimentally and in silico have proven effective. While these tools can predict the locations of enhancers with a high degree of accuracy, the mechanisms underpinning the activity of enhancers are often unclear.

**Results:**

Using machine learning (ML) and a rule-based explainable artificial intelligence (XAI) model, we demonstrate that we can predict the location of known enhancers in Drosophila with a high degree of accuracy. Most importantly, we use the rules of the XAI model to provide insight into the underlying combinatorial histone modifications code of enhancers. In addition, we identified a large set of putative enhancers that display the same epigenetic signature as enhancers identified experimentally. These putative enhancers are enriched in nascent transcription, divergent transcription and have 3D contacts with promoters of transcribed genes. However, they display only intermediary enrichment of mediator and cohesin complexes compared to previously characterised active enhancers. We also found that 10–15% of the predicted enhancers display similar characteristics to super enhancers observed in other species.

**Conclusions:**

Here, we applied an explainable AI model to predict enhancers with high accuracy. Most importantly, we identified that different combinations of epigenetic marks characterise different groups of enhancers. Finally, we discovered a large set of putative enhancers which display similar characteristics with previously characterised active enhancers.

**Supplementary Information:**

The online version contains supplementary material available at 10.1186/s13059-021-02532-7.

## Background

Regulation of gene expression in eukaryotic cells is a complex process governed by interactions between DNA binding proteins (transcription factors), and the regulatory elements in DNA to which they bind. Mutations in these non-coding regulatory elements can cause disease states by affecting the spatial and temporal control of gene expression [1–4]. Identification of regulatory regions and understanding their function and interactions with transcription factors is not only important to furthering our understanding of biological systems, but also for providing a better understanding of disease states.

*Cis*-acting DNA sequences that increase the transcription of one or more genes are called enhancers [5, 6]. Unlike promoters that are generally located proximally to the transcription start site [7], the position of an enhancer relative to its target gene is highly variable and can occur upstream, downstream, or within introns [6, 8]. To achieve regulation of distal target genes, enhancers must make 3D contacts with the promoters of genes that they control [9]. In addition to not having a specific location in the genome, there is no general sequence code for enhancers and a given enhancer may only be active only in specific spatial, temporal, or environmental conditions [10]. All of these features complicate the discovery and annotation of enhancers both experimentally and computationally.

Enhancers act as platforms for transcription factor binding and display high DNA accessibility. However, these regions also exhibit specialised histone modifications, both overlapping and flanking transcription factor binding sites [11–13]. Several histone modifications have been linked with enhancer activity in the past. H3K4me1 enrichment has been observed at enhancers and is one of the primary marks used in enhancer identification [14]. H3K4me3 has been mainly identified at active promoters but has also been linked with enhancer activity [15]. H3K27ac has been originally identified as a mark that is used to separate active enhancers from poised enhancers [16], but subsequent work has found that H3K27ac alone does not indicate enhancer activity [17–19]. H4K16ac has been associated with active enhancers in mouse embryonic stem cells and Drosophila cells but is also enriched around the TSS of active genes [19, 20]. In addition to these histone tail modifications, globular domain modifications such as H3K122ac have also been used to identify active enhancers that lack some classical marks of enhancer activity [21]. In addition to histone modifications, active enhancers are also characterised by the presence of a class of small RNAs called enhancers RNAs (eRNAs) [22]. While specific epigenetic marks associated with enhancer activity have been identified, there is no comprehensive combinatorial epigenetic code of enhancers.

Recent methodological advances have made the genome-wide detection of enhancers possible. Self-transcribing active regulatory region sequencing (STARR-seq) is a massively parallel reporter assay that is able to identify enhancers based on their genome-wide activity and provides a quantitative measure of the enhancer activity [23, 24]. This makes the experimental identification of enhancers easier but does not provide a complete list of enhancers and mechanistic understanding of why certain regions of the DNA act as enhancers, while others do not. Despite these improvements in the identification of enhancers through high throughput analysis, the specific combination of epigenetic factors that determine whether a given region will act as an enhancer are unclear.

Computational approaches and, in particular, machine learning (ML) methods have been applied successfully to the identification of enhancers [19, 25, 26]. These methods use histone modifications and massively parallel reporter assay for enhancer identification as training data. Despite their relative success, ML methods suffer from biases and seem to identify large numbers of promoters rather than only enhancers [19, 27]. ML methods (such as artificial neural networks and random forest models) are good at providing accurate predictions, but the rules and insights which are used to make these predictions remain unclear. Rule-based explainable AI (XAI) models that generate natural language “If/Then” rules are classification algorithms that can be used for identifying enhancers using ChIP datasets. By using an XAI method (based on Type-2 Fuzzy Logic and Multi Objective Multi Constraint Evolutionary Computation), the rules used to make predictions can be generated, interpreted, and subsequently tested for validity [28, 29]. This XAI model provides a set of understandable rules and linguistic labels which can be unpacked and studied to understand the relationships deemed important for enhancer activity. Therefore, this represents a method that can be used to overcome limitations of ML approaches.

Here, we apply ML driven XAI models to predict and disentangle the effect of different epigenetic modifications on enhancer activity. We train our model in Drosophila BG3 cells, using histone modifications ChIP and STARR-seq datasets, and use it to predict enhancers in a different Drosophila cell line, S2. To evaluate the performance, the XAI model is compared with a traditional Neural Network ML approach and annotation of enhancers by STARR-seq. Using this approach, we successfully trained an explainable model, that accurately predicts enhancer locations and generalises to other cell lines without adjustment. Our model also predicted a population of putative enhancers not previously annotated by STARR-seq which we further characterise and explore. Some of the predicted enhancers are longer than 1 Kb and resemble mammalian super enhancers [30]. Our main aim is to evaluate how successfully the model generalises to a cell line that it had not been trained on, and, thus, we only trained the model in BG3 cells. Nevertheless, the performance of the model in S2 cells, indicates, that the BG3 rules are generalisable and can explain enhancers in other cell types.

## Results

### ML and explainable AI can predict STARR-seq enhancers and identify a set of novel enhancers

**Fig. 1:**
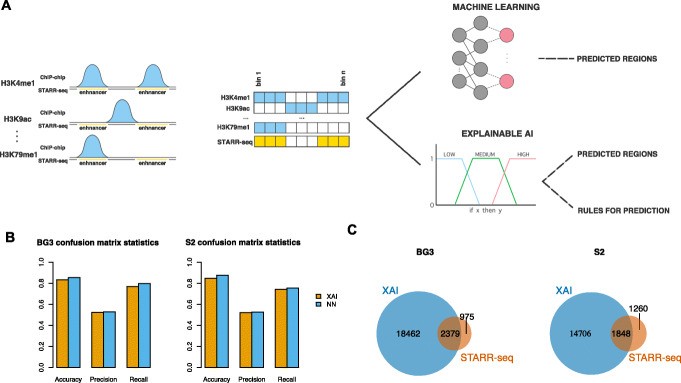
Explainable AI and machine learning models for enhancer identification. **A** A graphical representation of our rule based and machine learning analysis. ChIP-seq data for histone modifications and STARR-seq enhancer annotations are combined and tiled into bins covering the Drosophila genome. Using these bins, traditional machine learning models (ML) and explainable AI models (XAI) can be trained to predict enhancer locations. **B** Confusion matrix statistics from individual bin predictions. TP— true positives (detected by XAI/ML and STARR-seq), TN—true negatives (not detected by XAI/ML or STARR-seq), FP—false positives (detected only by XAI/ML), and FN—false negatives (detected only by STARR-seq). Accuracy ((TP + TN)/(TP + TN + FP + FN)), precision (TP/(TP + FP)), and recall (TP/(TP + FN)) were computed and plotted for the best performing explainable AI and neural network (NN) models. All models were trained in BG3 cells and then applied to S2 data. **C** Overlap between XAI and STARR-seq predicted enhancers in BG3 and S2 cells respectively

ChIP-seq data for histone modifications [31, 32] and STARR-seq enhancer annotations [23, 33, 34] are combined and tiled into bins covering the Drosophila genome (Fig. 1A). Using these bins, we trained a traditional machine learning model (neural network) and an XAI model to predict enhancer locations. The two trained models showed comparable accuracy (Fig. 1B). This demonstrates that XAI models display similar performance to neural networks, while providing the advantage of interpretation of the underlying results. To investigate how well the ML and explainable AI models generalise, we trained the models on data from BG3 cells and predicted enhancers in S2 cells using the corresponding histone modifications ChIP datasets (Fig. 1B). Although the models had not previously been exposed to data from S2 cells, it performed with a similar degree of accuracy, highlighting its ability to generalise to new cell lines in different tissues and developmental stages. Furthermore, we also trained a model on S2 cell data, but, while this model performed well in S2 cells, it had a very low accuracy in BG3 cells (Additional file 1: Fig. S1). This indicates that the S2 trained model did not generalise well, and, thus, we selected the model trained in BG3 cells for the subsequent downstream analysis.

For both BG3 and S2 cells, the precision was lower (Fig. 1B), indicating that our ML and AI models annotated more enhancers compared to STARR-seq. This is in line with our previous observations that STARR-seq does not generate a complete annotation of enhancers [35]. It is worthwhile noting that the plasmid used to generate the two STARR-seq dataset used in our analysis had a high false-negative rate [24].

For the XAI model, we selected bins with a probability threshold of 0.8 or higher (see Materials and Methods and Additional file 1: Fig. S2) and merged them into regions that represent the predicted enhancers. In both BG3 and S2 cells, our XAI model identified the majority of the STARR-seq enhancers and predicted many novel regions (14,000–18,000) as putative enhancers not previously identified by STARR-seq (Fig. 1C).

### Characterisation of the putative enhancers

We first investigated whether these putative enhancers were previously identified by other methods, by comparing the overlap between XAI enhancers, STARR-seq and Enhancer Atlas 2.0 catalogue [26]. Additional file 1: Fig. S3 confirms that most of the putative enhancers were not previously annotated by other methods (experimental or computational). Nevertheless, we also observed that our XAI enhancers have a higher overlap with STARR-seq compared to Enhancer Atlas 2.0. This can be explained by the fact that enhancers from Enhancer Atlas 2.0 need to be detected by at least two independent methods, and, thus, enhancers annotated only by STARR-seq and missed by other ML or experimental methods are not classified as enhancers.

**Fig. 2:**
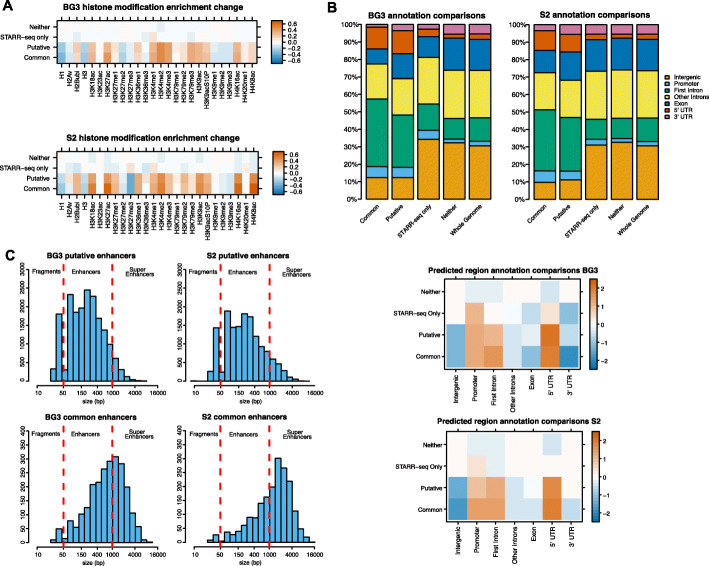
Characterisation of the putative explainable AI predicted enhancers. **A** log_2_(observed/expected) histone modification signal for different groups of regions: (i) enhancers detected by both STARR-seq and XAI (common enhancers), (ii) enhancers detected by XAI only (putative enhancers), (iii) enhancers detected by STARR-seq only, and (iv) regions detected by neither. Observed and expected values were computed using average normalised ChIP enrichment scores. **B** Top: Overlap between different groups of enhancers (see **A**) and genomic features: intergenic, promoter, first intron, other introns, exons, 5′UTR, and 3′UTR. Bottom: log_2_(observed/expected) overlaps based on whole genome distribution of the different annotations. **C** Histograms containing the size distribution for putative enhancers predicted by the XAI method in BG3 and S2 cells. Predicted enhancers shorter than 50 bp are considered fragments while regions above 1 kb are classed as super-enhancers

Enhancers which were detected by both the STARR-seq model and the XAI model were termed common enhancers and those that were detected only by the XAI model were termed putative enhancers (Fig. 1C). The putative enhancers display similar histone modifications to the common enhancers, namely: (i) strong enrichment of H3K18ac, H3K27ac, H3K4me1, H3K4me2, H3K4me3, H3K79me3, H3K9ac, H4K16ac and H4K8ac; (ii) partial enrichment of H2Bubi, H3K27me1, H3K36me1, H3K79me2, and H4K20me1; and (iii) depletion of H1, H3, and H3K27me2/3 (Fig. 2A). The depletion of histones (H1 and H3) and polycomb (H3K27me2/3) from enhancers was expected since active enhancers are located in regions of open chromatin. We only found negligible differences between the putative and common enhancers. Furthermore, the observed enrichment and depletion of alternative histone modifications at the putative enhancers suggest strong validity of these putative enhancers.

The enhancers detected solely by STARR-seq show only partial enrichment of H3K4me1 and very little enrichment or depletion of any other histone modifications. This explains why the machine learning and AI models could not identify these enhancers based on histone modification code. It is possible that either STARR-seq only enhancers might not act as true enhancers within the chromatin environment or that additional histone modifications that we did not include in our analysis characterise these enhancers (e.g. H3K56ac [36] or H3K122ac [21]).

We also investigated where these putative enhancers are located in comparison to common enhancers (also detected by STARR-seq). Our results confirm that the majority of putative enhancers and common enhancers are intronic (Fig. 2B). In addition, there is a specific overrepresentation of putative and common enhancers at 5’UTRs. These 5′UTRs regions that are annotated as enhancers may represent alternative promoters from the genes [37–39]. Since STARR-seq enhancers are also enriched at 5′UTR and we trained our model on STARR-seq data, it is not surprising that we predict these regions as enhancers.

Most putative enhancers fit the expected size based on previously identified enhancers (50 bp–1 Kb) (Fig. 2C). Nevertheless, we also identified larger regions that were classified as enhancers (more than 1 Kb) and we classified them as potential super enhancers [30]. It should be noted that common enhancers between XAI and STARR-seq tend to be longer. As the model predicts individual 10 bp bins, noisy regions in ChIP signals can lead to small false-positive regions being predicted as enhancers. Thus, enhancers shorter than 50 bp are likely to be artefacts, and in the downstream analysis, we only consider enhancers larger than 50 bp.

### Putative enhancers display 3D contacts with promoters of expressed genes

**Fig. 3:**
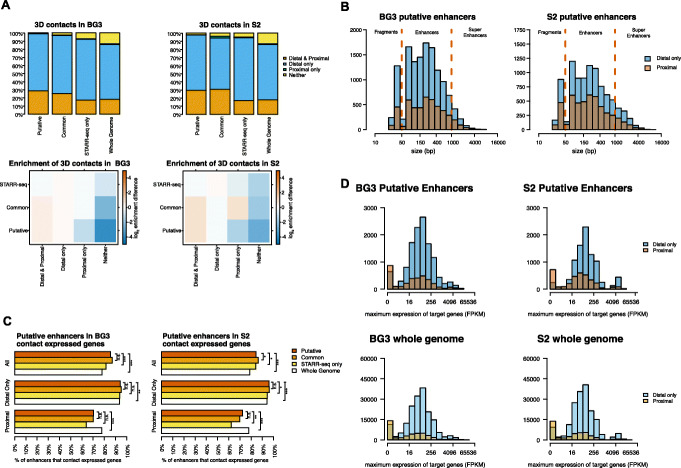
Putative enhancers make 3D contacts with expressed genes. **A** We split enhancers into the following: (i) proximal if they are located within 5 Kb of a promoter, (ii) distal if they are further than 5Kb from any promoters and make 3D contact with promoters, (iii) proximal only if they do not have enriched Hi-C contacts more than 5 kb away but were within 5 Kb of a promoter, and (iv) neither if they are further than 5Kb from any promoter and do not make 3D contacts with any promoters. Top: Putative, common, and STARR-seq enhancers have enriched 3D contacts with regions containing proximal (within 5 Kb from enhancer) or a distal (further than 5 Kb from the enhancer) promoters. We considered the case of BG3 and S2 cells respectively. Bottom: log_2_(observed/expected) based on whole genome distribution of the different annotations. **B** The size of distal only and proximal putative enhancers in BG3 and S2 cells on log_2_ scale. There is negligible difference between distal only or proximal putative enhancers (Mann-Whitney *U* test of log_2_ of size; *p* value < 1.34 × 10^−5^ for BG3 and *p* value = 0.09 for S2). **C** Majority of the enhancers that make 3D contacts with genes contact expressed genes, but there are significantly more distal only than proximal enhancers that contact expressed genes (Fisher’s extract test; *p* value: n.s. ≥ 0.05, *p value < 0.05, ** < 0.01 and *** < 0.001). **D** Top: Expression (FPKM) for proximal and distal only putative enhancers on log_2_ scale. We considered the maximum expression, in the case where promoters of multiple genes were contacted. There is a higher expression for genes controlled by distal only enhancers compared to proximal ones (Mann-Whitney *U* test of log_2_ of FPKM; *p* value < 2.2 × 10^−16^ for BG3 and S2). Bottom: Expression (FPKM) for proximal and distal only background regions on log_2_ scale. There is a higher expression for genes contacted by distal only background regions compared to proximal ones (Mann-Whitney *U* test of log_2_ of FPKM; *p* value < 2.2 × 10^−16^ for BG3 and S2). In BG3 cells, distal only enhancers have a mean log_2_ of FPKM of 6.08, while distal background regions of 5.82 (*p* value < 2.2 × 10^−16^). Similarly, in BG3 cells, proximal enhancers have a mean log_2_ of FPKM of 4.59 and proximal background regions of 3.77 (*p* value < 2.2 × 10^−16^). In S2 cells, distal only enhancers have a mean log_2_ of FPKM of 6.54, while distal background regions of 5.94 (*p* value < 2.2 × 10^−16^). Similarly, in S2 cells, proximal enhancers have a mean log_2_ of FPKM of 4.86 and proximal background regions of 3.81 (*p* value < 2.2 × 10^−16^). Note that in each case, we performed a Mann-Whitney *U* test

To further investigate whether these putative enhancers control gene expression, we integrated the putative, common and STARR-seq only enhancer annotation together with in situ sub-kilobase-pair resolution Hi-C datasets in BG3 [35] and S2 [40] cell lines with RNA-seq data [41]. Between 25 and 30% of putative and common enhancers are proximal (within 5 Kb) to a gene promoter, while approximately 70% are not within 5 Kb of promoters however do make 3D contacts with gene promoters that are located more than 5 Kb away (Fig. 3A). All proximal putative enhancers contact distal genes as well, indicating that majority of promoters come in 3D proximity to other promoters [42]. Only a negligible number (less than 2.1% in every cell line) are not proximal to a promoter or do not have 3D contacts with any distal promoter. In contrast, a significantly larger proportion of the genomic background (13.5% in BG3 and 13.1% in S2) are not proximal to any promoter or do not have 3D contacts with any distal promoter. The high number of 3D contacts between the genomic background and promoters (3.8 M in BG3 and 4.3 M in S2) can be explained by the fact that the genomic background contains many exons and introns (see Fig. 2B) and expressed genes in Drosophila form gene domains with many enriched 3D contacts [43].

There is a negligible difference in size between the distal only and proximal putative enhancers (Fig. 3B). Most importantly, a majority of putative enhancers (approximately 85%) make 3D contacts with promoters of transcribed genes (Fig. 3C). While approximately 70% of proximal putative enhancers sit within 5 Kb of promoters of transcribed genes, 94% of distal only putative enhancers contact promoters of transcribed genes. In addition, the distal only enhancers also tend to have 3D contacts with promoters of genes that display higher expression compared to proximal putative enhancers (Fig. 3D). Note that the genomic background has similar high proportions of contacted expressed genes, which indicates that once a region makes 3D contact with the promoter of a gene, the gene is often transcribed, especially when the region is distal to any promoters.

One possibility that could explain why the putative enhancers are not detected by STARR-seq is that these putative enhancers act mainly distally, while enhancers detected by STARR-seq mostly act proximally. We found that this is not the case and both common enhancers between XAI and STARR-seq (Fig. 3 and Additional file 1: Fig. S4) and STARR-seq only enhancers (Fig. 3 and Additional file 1: Fig. S5) make 3D contacts with expressed genes. In fact, STARR-seq only enhancers are less proximal to promoters of genes and have the highest proportion of distal 3D contacts with promoters (74.7% in BG3 and 77.4% in S2) (Fig. 3).

There also exists the possibility that these putative enhancers are redundant enhancers. To investigate this, we plotted the enriched Hi-C contacts of putative and common enhancers to all other predicted enhancers (including both putative and common) (Additional file 1: Fig. S6). Common enhancers tended to contact more enhancers, but whether this is due to the common enhancers themselves generally being larger or another mechanism is not clear.

### XAI provides explainable rules for annotation of enhancers

**Fig. 4:**
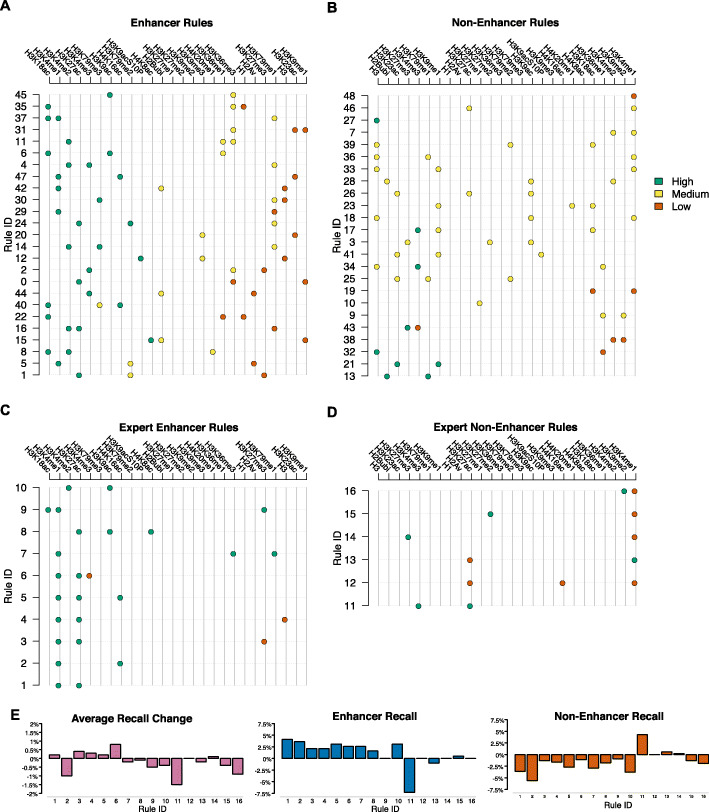
Rules explaining enhancer and non-enhancer classification. Individual rules are horizontal lines on the plot and include up to three epigenetic marks per rule. The colour code represents classification of an epigenetic mark as high (green), medium (orange), or low (red). **A** Rules involved in predicting bins as belonging to enhancers. **B** Rules that contribute to predicting bins not belonging to enhancers (non-enhancer). **C**, **D** expert rules for enhancers (**C**) and non-enhancers (**D**). **E** Evaluation of the expert rules. We plot the recall for enhancers and non-enhancers and the average recall.

Figure 4A, B shows the rules identified by our explainable AI model to classify regions as either enhancers or non-enhancers in Drosophila. The rules were determined to be the most effective while remaining explainable when constrained to a maximum of three epigenetic modifications per rule, and a maximum of 50 rules. These parameters were chosen to ensure that the model was explainable while maintaining a high degree of predictive power.

Individual classifications of epigenetic marks as low, medium, and high do not have stringent borders but instead allow for some overlap between classes. These boundaries vary by mark and are trained when training the model. Interestingly, the rules for enhancers contain high levels of H3K4me1 together with either high level of H4K16ac or H3K18ac, but not with high levels of H3K27ac (Fig. 4A). Nevertheless, 70% of the regions displaying high levels of both H3K4me1 and H3K27ac are selected by our explainable AI model, and, consequently, can be explained by other combinations of histone modifications. Interestingly, it was recently shown that H3K27ac is not required for enhancer activity and its depletion at enhancers in mouse ES cells results in only a few small changes in gene expression [18].

It is also interesting to note that in mouse ES cells H4K16ac has been found to mark active enhancers both with and without H3K27ac [20]. However, in Drosophila, it has been mainly associated with dosage compensation together with MOF [44, 45]. To avoid biases from dosage compensation effects, our models and predictions were run only on autosomes, which means that we can identify enhancers in Drosophila that are characterised by high levels of both H3K4me1 and H4K16ac, similar to those seen in mammalian systems. We found that approximately 9% of all predicted enhancers in BG3 cells are characterised by high levels of H3K4me1 and H4K16ac. One example is rule 47 which triggers when H3K4me1 enrichment is high, H3K16ac is high, and H2K23ac is low (Fig. 4A).

Figure 4B contains rules used to classify regions as non-enhancers, and, not surprisingly, non-enhancers are depleted in H3K4me1. For example, rule 19 states that if H3K18ac and H3K4me1 are low, then the region will be classified as a non-enhancer. Non-enhancers are also characterised by depletion of H3K4me2 and H3K9me2 or enrichment of H3, H3K27me3 and H3K4me3.

Interestingly, enrichment of H3K4me3 is usually associated with promoters [12], but broad H3K4me3 peaks have been previously associated with enhancers [46]. Our results show that when high levels of H3K4me3 are associated together with depletion of H3K27me3 or depletion of H2AV, then those regions are classified as enhancers (Fig. 4A). Furthermore, high levels of H3K4me3 together with medium levels of H3K36me1 or H3K18ac characterised regions that are not classified as enhancers (Fig. 4B).

Using the XAI approach allows us to test expert rules. These are rules that are not generated by the multi objective and multi constraint evolutionary computation based genetic algorithms when building the model, but which experts in the field considers to be true. We tested if adding an expert rule “high levels of both H3K4me1 and H3K27ac define enhancers” improves the model, but we found only negligible improvement in predictions (less than 0.25%) (Fig. 4C–E). The expert rule that improved the predictions most (by approximately 1%) was that “high levels of both H3K4me1 and H3K27ac and low levels of H3K4me3 define enhancers”.

### Epigenetic code of developmental and housekeeping enhancers

**Fig. 5:**
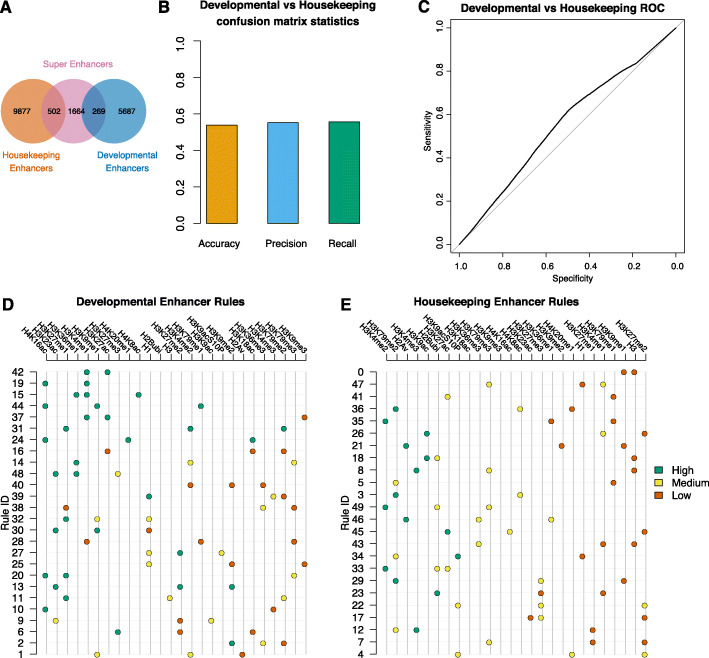
Training an XAI model to distinguish between housekeeping and developmental enhancers. **A** The developmental and housekeeping enhancers used in the training step and their overlap with predicted super enhancers. **B** Confusion matrices statistics from individual bin predictions. Accuracy, precision, and recall were computed and plotted for the XAI model (see Fig. 1). **C** ROC curve for developmental vs housekeeping trained model. **C**, **D** Rules involved in predicting bins as belonging to developmental enhancers (**C**) and housekeeping enhancers (**D**); see Fig. 4A, B

Housekeeping enhancers are enhancers expressed across multiple cell types and are crucial to the correct functioning of the cell, while developmental enhancers are enhancers that are cell type specific and are involved in differential gene expression across various cell types. Previous studies investigated whether different histone modifications are present at housekeeping and developmental enhancers, and it was proposed that developmental enhancers are devoid of histone modifications [47]. However, further research found preferential enrichment of H3K4me1 at developmental enhancers and H3K4me3 at housekeeping enhancers [48]. Using the annotation of housekeeping and developmental enhancers in S2 cells [34], we created two datasets of enhancers, housekeeping (10,379) only and developmental (5956) only with less than 5% being super-enhancers (Fig. 5A). Then, we used these annotations to train XAI models using the same epigenetic marks as before. Interestingly, we found that the model could not distinguish well between housekeeping and developmental enhancers based on histone modifications (AUC = 0.55) (Fig. 5B, C). Analysing the rules, we found that indeed H3K4me2/3, H3K79me2, and H3K9ac are more predictive of housekeeping enhancers and H3K4me1, H4K16ac and H3K27ac are more important for developmental enhancers (Fig. 5D, E). Interestingly, the rule “high levels of H3K4me1 and H3K27ac” was able to only negligibly improve the model performance for our enhancer annotation (Fig. 4C), but is associated with developmental enhancers and not housekeeping enhancers (Fig. 5D). Altogether, our results support a model where there are marginal epigenetic differences between housekeeping and developmental enhancers, but these differences were not sufficient to properly distinguish between the two.

### Enrichment of proteins and epigenetic factors at different classes of enhancers

**Fig. 6:**
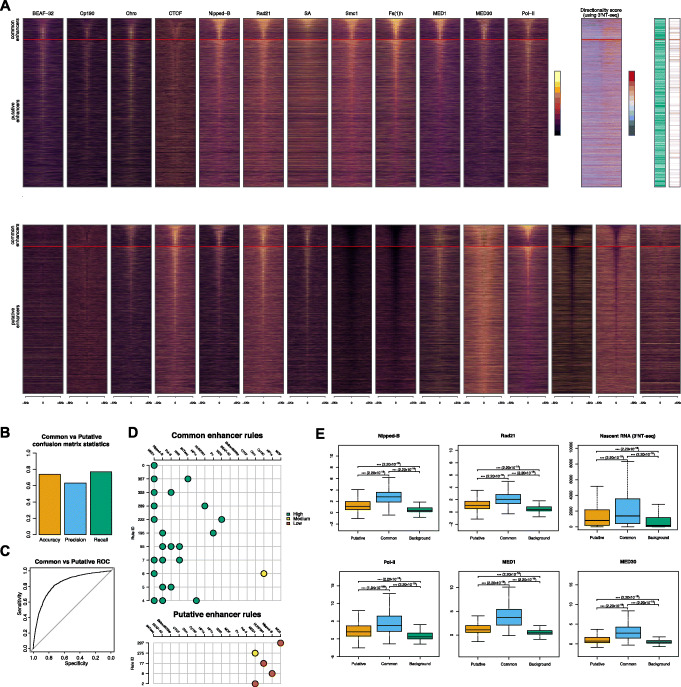
Enrichment of chromatin features at enhancers in BG3 cells. **A** We ordered enhancers based on their size (starting with largest at the top) and split them in common and putative enhancers. We plot profiles ± 5 Kb around the centre of enhancers for: architectural proteins (BEAF-32, Cp190, Chro, CTCF, Nipped-B, Rad21, SA, Smc1, Fs(1)h, MED1 and MED30), transcription (Pol II and 3'NT-seq), polycomb and heterochromatin (Pc, HP1a and HP1c), nucleosome remodelling (ISWI, MOF, WDS and NURF301), epigenetic factors (H3, H4, H3K4me3, H3K4me1, H3K27ac, H3K36me3, H3K79me1 and H4K16ac). We used 3′NT-seq to call bidirectional enhancers (see Materials and Methods), marked by a green bar, and also plot the directionality score as the log_10_ ratio of the nascent RNA signal on the positive and negative strands (with red indicating higher expression on positive strand and blue higher expression on the negative strand). We also marked by purple if there are any TAD borders within 2 Kb, using TAD borders annotation in BG3 cells from [35]. **B** Confusion matrices statistics from individual bin predictions. Accuracy, precision, and recall were computed and plotted for the XAI model (see Fig. 1). **C** ROC curve for common vs putative trained model. **D** All dominance 3 and higher rules generated after training a model to identify common vs putative enhancers. Individual rules are horizontal lines on the plot and include up to three epigenetic marks per rule. The colour code represents classification of an epigenetic mark as high (green), medium (orange), or low (red). **E** The 95th percentile score across the body of each common and putative enhancer was plotted. Mann-Whitney *U* test scores can be found on each plot for each group comparison

The two groups of enhancers, common and putative, were independently ordered based on their size. Following this, the distribution of protein and epigenetic marks around the two groups were plotted. Figure 6A shows that the majority of enhancers in BG3 cells (except some smaller ones) are characterised by the presence of BEAF-32, Cp190, and Chro. These three architectural proteins have been shown to be involved in 3D chromatin organisation and chromatin looping in Drosophila [35, 49–51], thus confirming the enrichment of 3D contacts with gene promoters observed. CTCF is enriched mainly at borders of larger common and putative enhancers suggesting insulation function [52]. Furthermore, we also observed enrichment of cohesin, mediator complex, and Pol II together with divergent transcription and lower levels of H3 and H4 (see Fig. 6), which are all common characteristics of active enhancers [38, 53]. While only larger common and putative enhancers are characterised by high levels of H3K27ac and H3K4me3, the majority of enhancers display high levels of H3K4me1 (see Fig. 6). This could be explained by the fact that larger enhancers could also harbour promoters with higher levels of H3K27ac and H3K4me3.

Most enhancers are also characterised by high levels of ISWI and NURF301 (nucleosome sliding), WDS (involved in maintenance of H3K4me3), and MOF (enzyme responsible for acetylating H4K16) together with high levels of HP1c (see Fig. 6A). ISWI and MOF are involved in nucleosome remodelling and are important to maintain active chromatin marks and, thus, are expected to be identified at enhancers [54, 55]. The latter (HP1c) is enriched in euchromatin, explaining its localisation at predicted enhancers, and, while it is closely related to HP1a, it does not seem to be involved in phase separation [56]. Interestingly we observed HP1a depletion inside the enhancers and its partial enrichment at the borders of larger enhancers, suggesting a potential mechanism for enhancer and promoter hubs.

Enhancers are often characterised by active transcription, usually divergent transcription [57–60]. We observed an enrichment of 3’NT-seq signal across both common and putative enhancers (Fig. 6A), suggesting that both groups of enhancers are transcriptionally active. Furthermore, both common and putative enhancers display strong bidirectional transcription (Fig. 6A), a characteristic of enhancers [57, 58]. What differentiates putative and common enhancers is the level of nascent RNA displayed, with putative enhancers having an intermediary level of nascent RNA, while common enhancers have the highest level of nascent RNA (Fig. 6E). This result is further mirrored by Pol-II signal, which is higher at common enhancers than at putative ones (Fig. 6E). Altogether, our results show that while putative enhancers display activity, this is lower than the activity of common enhancers, which can potentially explain why they were not detected with the original STARR-seq plasmid.

To further evaluate potential subtle differences between the common and putative enhancers, we trained an additional XAI model to predict whether a given enhancer is common or putative based on features in Fig. 6A. The model we trained was able to predict which group an enhancer belonged to with a 77.1% average recall and 0.84 AUC using a set of 16 features (Fig. 6B, C). Due to the similarities in function of subunits of cohesin and the mediator complex we used a single track for each of the two complexes (Nipped-B for cohesin and MED1 for Mediator) to prevent rules involving cohesin or the mediator complex from becoming diluted across several features. In particular, Nipped-B and SA recruit cohesin to the genome and Rad21 and Smc1 are two cohesion subunits [61]. Fs(1)h was found to recruit Nipped-B [62] and, thus, was also not included in the selected features. Mediator (MED1) appeared in 8 of the 11 and cohesin (Nipped-B) in 5 out of 11 high dominance rules predicting common enhancers. These features show a similar distribution between common and putative enhancers, but putative enhancers have a reduced intensity across these tracks (Fig. 6A). Furthermore, we plotted the distributions of all these features at common, putative, and background regions (Fig. 6E and S7). Our results show that all the cohesin and mediator subunits display higher enrichment at common enhancers than at putative ones, but these features are still more enriched in putative enhancers than they are across the genomic background. We hypothesise that the reduced recruitment of cohesin and mediator complex at these enhancers may prevent them to display similarly high activity as common enhancers. Furthermore, other architectural proteins (BEAF-32, Cp190, Chro and CTCF) or chromatin remodellers (ISWI, MOF, WDS and NURF301) show similar strong enrichment at common enhancers and medium enrichment at putative enhancers (Additional file 1: Fig. S7).

Previously, we found that BG3 specific TAD borders display enhancer like epigenetic landscape but were not identified as enhancers by STARR-seq [35]. Furthermore, the enrichment of architectural proteins and common and putative enhancers raised the question whether these enhancers are associated with TAD borders or not. Nevertheless, we found that only a small number of common and putative enhancers are located within 2 Kb of TAD borders (Fig. 6A).

Combinations of different chromatin modifications can be clustered, and, using Hidden Markov Models, a chromatin state map of the cell can be generated [31]. We used a recent 11 state chromatin state map for BG3 cells [36] and investigated the overlap of our predicted enhancers with the different chromatin states (Additional file 1: Fig. S8). Both common enhancers (detected by both STARR-seq and XAI) and putative enhancers (XAI specific) are mostly enriched in enhancer, active TSS, and active intron states and depleted in heterochromatin, polycomb, and basal. The only difference between common and XAI only enhancers is that the former displays slightly stronger enrichment in enhancer state and slightly stronger depletion in heterochromatin state. Enhancers detected by STARR-seq only are enriched only in competent state explaining why the XAI model did not classify them as enhancers.

Finally, to complement the analysis of enriched architectural proteins, we investigated if there are any transcription factors (TFs) that are preferentially enriched at common and putative enhancers. Additional file 1: Fig. S9A-B compares enrichment for known Drosophila TFs between common and putative enhancers in BG3 cells and S2 cells and shows that majority of enriched TF motifs are shared between common and putative enhancers. There are, however, several TFs motifs that are preferentially enriched at putative enhancers (55 in BG3 cells and 17 in S2 cells) and few that are enriched at common enhancers (21 in BG3 and S2); see Additional file 1: Table S1. We next compared how many of these are common between the two cell lines and found that 5 TF motifs are enriched in both cell lines specifically at putative enhancers (Mes2, dl, Asciz, ERR and USP) and 5 are enriched specifically at common enhancers (cnc maf-S complex, eg, Atf-2, tj and hkb) (Additional file 1: Fig. S9C-F).

Our explainable AI model uncovers a new set of putative enhancers, previously not identified in two Drosophila cell lines. Using experimental data, we show that these putative enhancers display similar epigenetic characteristics to enhancers detected by STARR-seq and make 3D contacts with transcribed genes. Altogether, we could not identify any significant difference in chromatin and epigenetic modifications between the putative and common enhancers suggesting that these putative enhancers are a novel group of previously uncharacterised enhancers.

## Discussion

High-throughput enhancer assays (such as STARR-seq or other massively parallel reporter assays) have revolutionised the identification of enhancers, but they also suffer from false positives and false negatives [23, 24, 63–65]. In addition, these methods are resource intensive, and it is not expected that we will have a STARR-seq genome-wide annotation of enhancers in every cell, tissue, or disease condition, especially in very large and complex organisms. For example, to address the difficulty and prohibitive cost of applying the method genome-wide, STARR-seq can be applied to regions of the genome where epigenetic marks specific to enhancers are found [66]. In addition, massively parallel reporter assays approaches only annotate enhancers and do not provide insights on why those genomic regions were identified as enhancers. Computational approaches can complement massively parallel reporter assays to annotate enhancers, but to address these issues, they need to be generalizable and explainable. The former (generalisation) ensures that once a model is trained in a cell line, tissue, or state, it can be applied to other cell types, tissues, and conditions without affecting the accuracy of the predictions. The latter (explainability) ensures that the rules used to classify enhancers can be accessible and interpreted.

In this manuscript, we use Opaque Box ML based Neural Networks and XAI (based on Type-2 Fuzzy Logic and Multi Objective Multi Constraint Evolutionary Computation) models to identify enhancers using STARR-seq enhancer annotation and a set of epigenetic features (e.g. histones and histone modifications). We train the model on a subset of regions in one cell line (BG3) and predict the enhancers genome wide in the same cell line and a different cell line (S2), which the model has not been exposed to during the training. Our results confirm that both the Opaque Box ML and XAI are able to predict enhancers with high accuracy and, most importantly, that they are generalizable (see Fig. 1), demonstrating the ability of the model to predict enhancers in alternative cell types that were not used to train the model.

Interestingly, we observed a decrease in the precision of these computational models, meaning that they predict more enhancers than those annotated by STARR-seq. The critical question is whether these putative enhancers are true enhancers or not. We know that STARR-seq recovered enhancers are dependent on the plasmid used in the experiment, suggesting that the plasmid used could lead to an increase in false negatives (true enhancers that are missed) [24]. In particular, the STARR-seq datasets we used in this study were generated using an older version of the plasmid that has been shown to miss enhancers [24]. In addition, some of these putative enhancers (approximately 2500), albeit not all of them, have been previously characterised as enhancers by other methods in Enhancer Atlas 2.0 [26] (Additional file 1: Fig. S3). To validate whether these computationally predicted putative enhancers are true enhancers, we designed a series of tests. First, putative enhancers share the same epigenetic code as enhancers identified by STARR-seq (enrichment and depletion of same histone modifications). Furthermore, majority of these putative enhancers make 3D contacts with expressed genes. It is known that BEAF-32, Chro, and Cp190 are involved 3D chromatin interactions [35, 49, 50, 67]. The observed enrichment of these proteins at our predicted enhancers (Fig. 6) and that our predicted enhancers have 3D contacts with promoters provides a model of how the 3D promoter-enhancer interactions are mediated. Interestingly, both Pol II and divergent transcription were observed at these enhancers (Fig. 6). These are features that were previously identified as characteristic of active enhancers [38, 53], and their enrichment at our predicted enhancers provides further evidence of the validity of our predicted enhancers.

Enhancer hubs have been previously observed in many organisms and there is evidence that these redundant enhancers have a role in providing robustness in gene activation [68, 69]. Here, we found that putative enhancers display high number of enriched contacts with other enhancers, albeit less than in the case of common enhancers (Additional file 1: Fig. S6). One possibility is that our putative enhancers are potentially redundant enhancers that are part of enhancer hubs.

The main difference between the putative enhancers and the ones identified by both STARR-seq and the XAI models is that the putative enhancers tend to be slightly smaller (many of them are between 150 bp and 1Kb). Nevertheless, their size is within the expected size of enhancers [70, 71]. Enhancers detected by both STARR-seq and XAI are longer (larger than 1 Kb) and could be classified as super enhancers [30] or stretch enhancers [72]. Super enhancers have not previously been observed in Drosophila, and, due to their size, they are difficult to verify experimentally. However, the marks used to predict enhancer activity in this model correspond to the marks expected in either large enhancer group [73].

Furthermore, putative enhancers display slightly less enrichment in enhancer chromatin state and slightly less depletion in heterochromatin chromatin state compared to common enhancers. This indicates that while the putative enhancers share the same epigenetic code as common one, they might have less enrichment of the active chromatin marks. One possibility is that these putative enhancers are not as strong as the common ones and they might be missed by experimental methods. Alternatively, they might be primed enhancers [74], enhancers displaying most of the active marks, but not activating transcription yet. Based on our analysis, we cannot exclude that some of the putative enhancers are primed enhancers.

### Epigenetic code of enhancers

One of the advantages of XAI is that we can see and evaluate the rules used to predict enhancer activity. Some rules are well described in the literature, for example H3K4me1 or H3K27ac being highly enriched [14, 16]. Other rules were more surprising, for example, H3K23ac has not been extensively studied but it appears to be one of the stronger negative predictors of enhancer activity according to the model, appearing in several rules with a very distinct pattern. Furthermore, we found that 9% of enhancers display enrichment of H4K16ac that, in Drosophila, it has been mainly associated with dosage compensation [44, 45]. Nevertheless, H4K16ac has been found at active enhancers in a mammalian cell line independent of the presence of H3K27ac [20]. In addition, H3K9ac has been shown to be an important histone modification located at strong active enhancers in Drosophila [75], and it appears in two of our detected enhancer rules. Finally, we also observed enrichment of H3K18ac at our predicted enhancers and this mark is known to be enriched at enhancers in Drosophila [31].

Interestingly, we found that there is no rule in our XAI model where both H3K4me1 and H3K27ac are high (see Fig. 4), despite these marks often being used as a proxy for enhancer identification (reviewed in [12]). This was puzzling at first so we therefore investigated how many of our predicted enhancers have high levels of H3K4me1 and H3K27ac. Interestingly, 70% of our enhancers are characterised by H3K4me1 and H3K27ac ChIP peaks and adding an expert rule of “enhancers are characterised by high levels of H3K4me1 and H3K27ac” only improves the model marginally (0.25%). This suggests that our model captures the regions containing high levels of H3K4me1 and H3K27ac by different combinations of histone modifications, which could be potentially more selective.

One important question is whether the epigenome can be used to differentiate between developmental and housekeeping enhancers. Previous studies have provided contradictory results, and, while one study found that there is very little epigenetic signal [47], a different study found preferential enrichment of H3K4me1 at developmental enhancers and H3K4me3 at housekeeping enhancers [48]. Our results support both of these findings, and while we found that indeed H3K4me2/3 is more important for housekeeping enhancers and H3K4me1 for developmental enhancers, the epigenetic codes of housekeeping and developmental enhancers are very similar and there is not enough information to distinguish clearly between the two.

### Associated proteins at enhancers

BEAF-32, Chro, and Cp190 have all been found to be strongly enriched around distal and proximal enhancers, but this was more prevalent at housekeeping enhancers [48]. We found the enrichment of these architectural proteins at majority of the predicted enhancers (Fig. 6). CTCF is known to have insulation functions [52] and its presence at the borders of larger putative and common enhancers, which indicates that it plays a role in halting the spread of heterochromatin inside enhancers. Interestingly, HP1a is also enriched at the borders of these enhancers suggesting that heterochromatin is potentially phase separated in order to keep it away from enhancers [56].

Our results show medium enrichment of cohesin (Rad21, Smc1, Nipped-B and SA) and architectural proteins (BEAF-32, Cp190, Chro and CTCF) [35, 76] at putative enhancers. This is in contrast with the higher enrichment of these proteins at common enhancers (Fig. 6 and Additional file 1: Fig. S7). The lower enrichment of cohesin at putative enhancers correlates with lower activity (nascent RNA) that can lead to the enhancer displaying an activity below the detection threshold of high throughput enhancer assays (such as STARR-seq or other massively parallel reporter assays). Interestingly, recent studies have shown that cohesin is required for enhancer activity in mammalian systems, but mainly for long range enhancer activity [77, 78]. We find that the 3D genome architecture might play a role in enhancer activity in Drosophila similarly to mammalian system [61].

Our aim was to investigate if histone modifications are sufficient to predict enhancers and we did not include TFs in our predictive models. We found that indeed histone modifications are not only sufficient to recover most of the previously annotated enhancers by STARR-seq, but we also predicted many new putative enhancers. To investigate whether TFs could differentiate between common and putative enhancers, we tried to identify if there are any enriched motifs at the two classes of enhancers, and we found that majority of motifs are shared between the two groups of enhancers. Nevertheless, there were five TFs that were enriched preferentially at common in both BG3 and S2 cells (cnc maf-S complex, eg, Atf-2, tj and hkb) and five TFs that were enriched preferentially at putative in both BG3 and S2 cells (Mes2, dl, Asciz, ERR and USP). Only eg, hkb, Mes2, and maf-S are expressed in larval central nervous system (from where BG3 cells are derived), and only dl is expressed in S2 cells [79]. This suggests that there is only a negligible difference in enriched TF motifs between common and putative enhancers, and most of those differentially enriched TFs are not expressed in these two cells.

## Conclusions

Here, we use opaque box machine learning (neural networks) and explainable AI (based on Type-2 Fuzzy Logic and Multi Objective Multi Constraint Evolutionary Computation) models to successfully identify enhancers based only on epigenetic features and using STARR-seq enhancer annotation for training. Our results confirm that both the opaque box machine learning and explainable AI models can predict enhancers with a high degree of accuracy and, most importantly, that both models were able to generalise to a previously unseen cell line. We identify a novel set of putative enhancers that display a similar epigenetic landscape as enhancers identified by STARR-seq, but only intermediary levels of mediator and cohesin complexes and nascent transcription that are all significantly above background levels. Most importantly, we were able to analyse the rules employed by the explainable AI to identify enhancers and dissect the combinations of different histone modifications that characterise different classes of enhancers.

## Methods

### Datasets to train Opaque Box ML and XAI models

The Drosophila melanogaster genome (dm6) [80, 81] was tiled into bins of 10 base pairs (bp) with the sex chromosomes removed to eliminate potential biases arising from marks involved in dosage compensation mechanisms [44]. ChIP-chip datasets generated and pre-processed (M values smoothed over 500 bp) by the modENCODE Consortium for histone modifications and histone variants in two *D. melanogaster* cell lines (BG3 and S2) were downloaded from modEncode [82]. The full list of datasets used can be found in Additional file 1: Table S2. Enrichment scores from these datasets were transformed with min-max normalisation and then mapped into bins in the tiled genome. Self-transcribing active regulatory region sequencing (STARR-seq) datasets for BG3 and S2 cells were obtained from [23,3 3, 34]. STARR-seq peaks were expanded to 400 bp and then mapped to the 10 bp bins of the tiled genome.

### Opaque BoxML and XAI models

One million bins were sampled, maintaining the same ratio of enhancer to non-enhancer labelled bins found across the entire dataset. The data set was then split into training and testing datasets where all maintained the ratio of enhancers to non-enhancers. Using the Temenos XAI platform (https://logicalglue-support.helpscoutdocs.com/), neural networks, and type-2 fuzzy logic based XAI models were trained using the binarised STARR-seq peaks as classification labels for active enhancers. The best fitting models for each classification method were selected based on their accuracy, recall, and gini (gini =2 *area under the curve (AUC)-1) scores across the testing and the overall one million initially uploaded bins. These models were then used to predict enhancer activity across the previously tiled dm6 genome in both cell types, and their performance was compared.

Regions predicted to be enhancers using the XAI model were selected to have a probability threshold of 0.8 or higher based on Additional file 1: Fig. S2. These regions were compared to neighbouring regions within 100 bp upstream or downstream. If the combined regions average probability threshold was above 0.8, the bins were merged; if not, the regions were kept separate.

### 3D enriched contacts and expression data

The 3D contacts of these potential enhancers were then explored using Hi-C data generated from [35, 40]. The enriched contacts were extracted with HiCExplorer using the observed/expected ratio method [51]. Promoter locations were defined as being 250 bp upstream of TSS sites from the dm6 GFF3 annotation [81]. Enhancers occurring in Hi-C bins with enriched contacts with regions containing promoters were classed as having potential promoter 3D contacts. Where these contacts were within 5 Kb, the contact was classed as proximal, and where contacts were outside of 5 kb, these were classed as distal.

Expression data (FPKM) from [41] was then examined for genes where the gene’s promoter made 3D contact with a potential enhancer.

### Architectural proteins and transcription and TF enrichment at enhancers

We used the ChIP-chip datasets generated and pre-processed (*M* values smoothed over 500 bp) by The modENCODE Consortium for BG3 cell lines [31, 32, 83, 84]. The NippedB ChIP dataset was downloaded from [61]. The full list of datasets used can be found in Additional file 1: Table S2.

For ChIP-chip occupancy analysis, we sorted the enhancers from the largest (top) to shortest (bottom) and extracted the ChIP-chip signal within 10 Kb window build around the enhancer centres. Initial profiles were winsorised (excluded all negative signals and everything above 95% quantile of positive signals) and rescaled to lie between 0 and 1. For 3′NT-seq profile, enhancers were sorted analogically, i.e., we did not apply the winterisation, and we rescaled positive and negative signals separately—positive signals were rescaled to the interval from 0 to 1; negative signals were rescaled to the interval from − 1 to 0. The signals of resulting profile belong to the interval from − 1 to 1.

The directionality score computed as log_10_ of the ratio between nascent RNA levels in 500 bp on the positive strand downstream of the border and on the negative strand upstream of the border. 500 bp bins that were 500 bp away were considered in both directions from the enhancer [35]. The directionality score was sorted analogically to the ChIP-chip profiles with respect to the enhancer sizes. The borders with directionality score between − 0.5 and 0.5 were treated as bidirectional and were coloured in green. Non-transcribed, positively transcribed, and negatively transcribed enhancers were coloured in white. In Fig. 6, we also marked by purple the enhancers that were located within 2 Kb of TAD borders in BG3 cells [35].

PWM enrichment analysis was performed on putative enhancer regions using the PWMenrich package in R [85] using MotifDb collection of TF motifs [86]. Significantly enriched TF motifs were classed as those having a *p* value of < 0.05 in PWMenrich when compared to the standard dm6 background.

## Supplementary Information

**Additional file 1:.** Supplementary Figures and Tables

**Additional file 2:.** Review history

## Data Availability

The full list of datasets used can be found in Additional file 1: Table S2. The scripts used for this analysis can be found at https://github.com/JC-Wolfe/XAI [87, 88] under the GPL3.0 license.
