# Supplementary material for: An explainable artificial intelligence approach for decoding the enhancer histone modifications code and identification of novel enhancers in Drosophila
Source: Genome Biol. 2021 Nov 8;22:308. doi: 10.1186/s13059-021-02532-7 (PMC8574042; doi:10.1186/s13059-021-02532-7)
Supplement: Supplementary file 1 — Additional file 1:. Supplementary Figures and Tables [file 13059_2021_2532_MOESM1_ESM.pdf]

Additional file 1 - Supplementary Information

An explainable artificial intelligence approach for decoding the enhancer histone modifications code and identification of novel enhancers in Drosophila

Jareth C. Wolfe[1,2,4], Liudmila A. Mikheeva[1,3,4], Hani Hagras[2,*], Nicolae Radu Zabet[1,4,*]
[1] School of Life Sciences, University of Essex, Colchester, CO4 3SQ, UK
[2] School of Computer Science and Electronic Engineering, University of Essex, Colchester, CO4 3SQ, UK
[3] Department of Mathematical Sciences, University of Essex, Colchester, CO4 3SQ, UK
[4] Blizard Institute, Barts and The London School of Medicine and Dentistry, Queen Mary University of London, London, E1 2AT, UK
[*] Corresponding authors r.zabet@qmul.ac.uk and hani@essex.ac.uk

Supplementary Figures

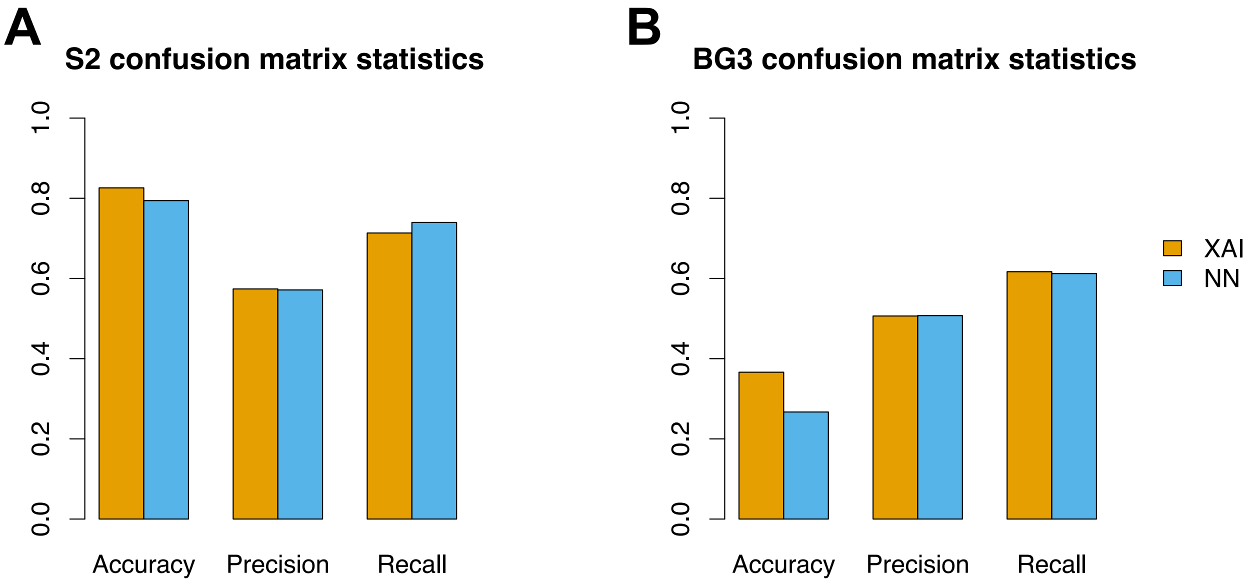

Fig. S1. *Confusion matrix statistics from individual bin predictions for the S2 trained model.* TP - true positives (detected by XAI/ML and STARR-seq), TN - true negatives (not detected by XAI/ML or STARR-seq), FP - false positives (detected only by XAI/ML) and FN - false negatives (detected only by STARR-seq). Accuracy ((TP + TN)/(TP + TN + FP + FN)), Precision (TP/(TP + FP)) and Recall (TP/(TP + FN)) were computed and plotted for the best performing explainable AI and neural network (NN) models. We applied the S2 trained model in (A) S2 cells and (B) BG3 cells.

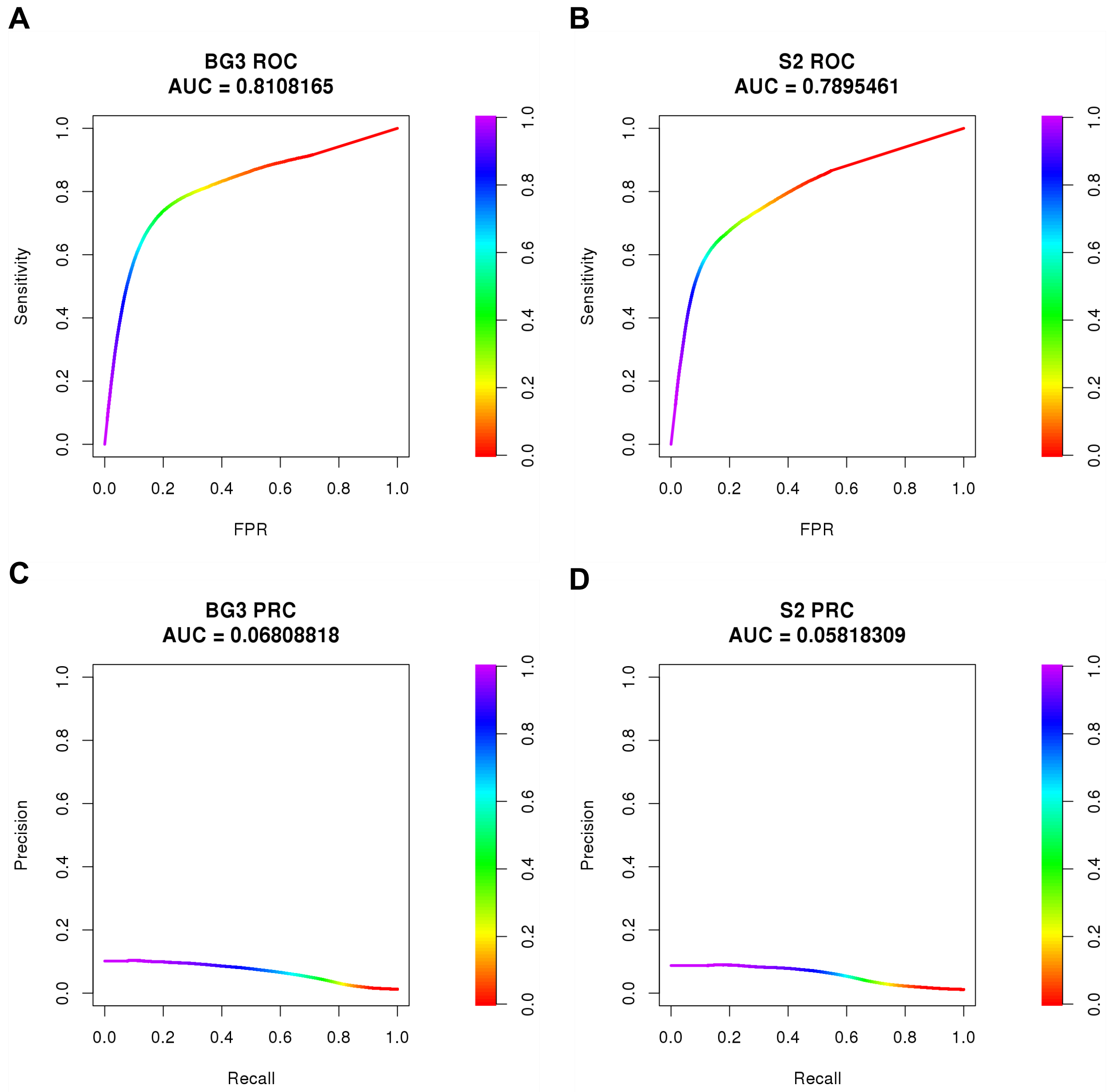

Fig. S2. *Performance of the XAI model.* (A and B) ROC curves for (A) the BG3 trained model and (B) the S2 trained model. (C and D) The PR curves for (C) the BG3 trained model and (D) the S2 trained model. Colour represents the threshold values used to compute the corresponding False Positive Rate (FP/(FP+TN)), Sensitivity (TP/(TP+FN)), Recall (TP/(TP + FN)) and Precision (TP/(TP + FP)); where, TP - true positives, TN - true negatives, FP - false positives and FN - false negatives.

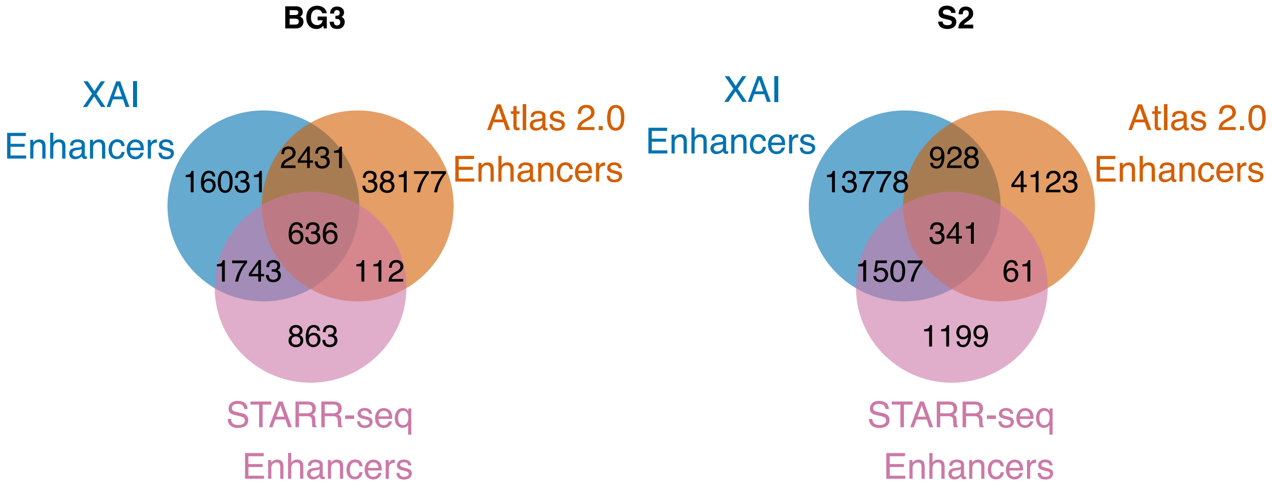

Fig. S3. *Overlap of XAI enhancers with STARR-seq and Enhancer Atlas 2.0.* We consider the case of BG3 and S2 cells separately.

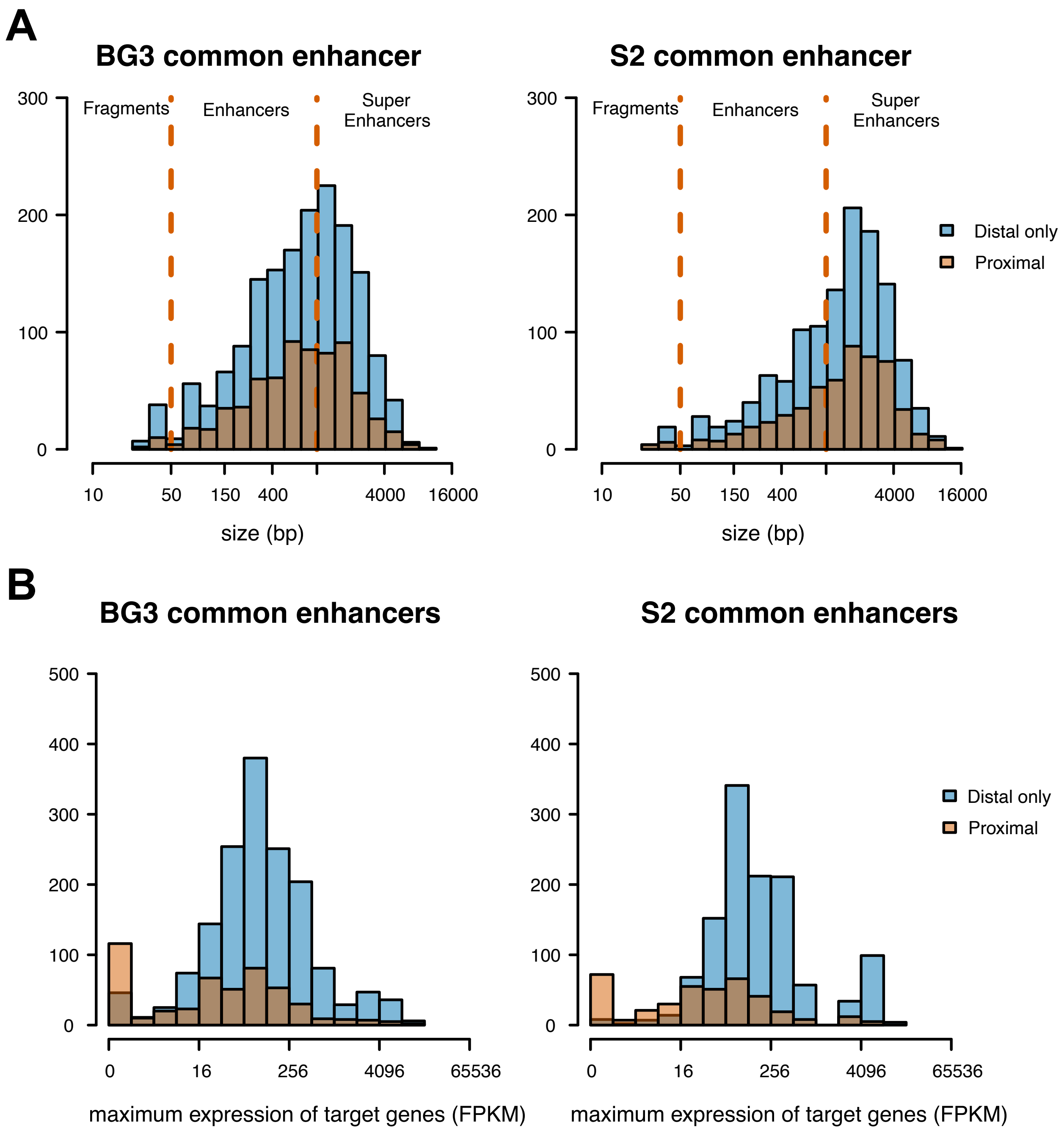

Fig. S4. *Common enhancers make 3D contacts with expressed genes.* (A) The size of distal only and proximal common enhancers in BG3 and S2 cells on $\log_2$ scale. There is negligible difference between distal only or proximal common enhancers (Mann-Whitney U test of $\log_2$ of size; p-value = 0.52 for BG3 and p-value = 0.32 for S2). (B) Expression (FPKM) for proximal and distal only common enhancers on $\log_2$ scale. We considered the maximum expression, where promoters of multiple genes were contacted. There is a higher expression for genes controlled by distal only enhancers compared to proximal ones (Mann-Whitney U test of $\log_2$ of FPKM; p-value $< 2.2\times10^{-16}$ for BG3 and S2).

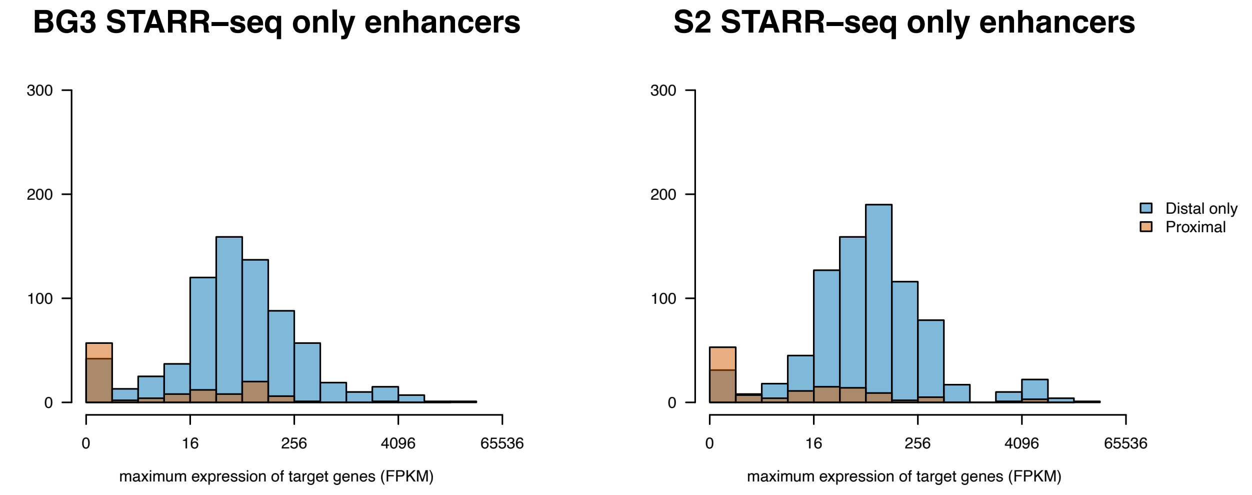

Fig. S5. *STARR-seq only enhancers make 3D contacts with expressed genes.* Expression (FPKM) for proximal and distal STARR-seq only enhancers on log_2 scale. We considered the maximum expression, in the case where promoters of multiple genes were contacted. There is a higher expression for genes controlled by distal only enhancers compared to proximal ones (Mann-Whitney U test of log_2 of FPKM; p-value $< 2.2\times10^{-16}$ for both BG3 and S2).

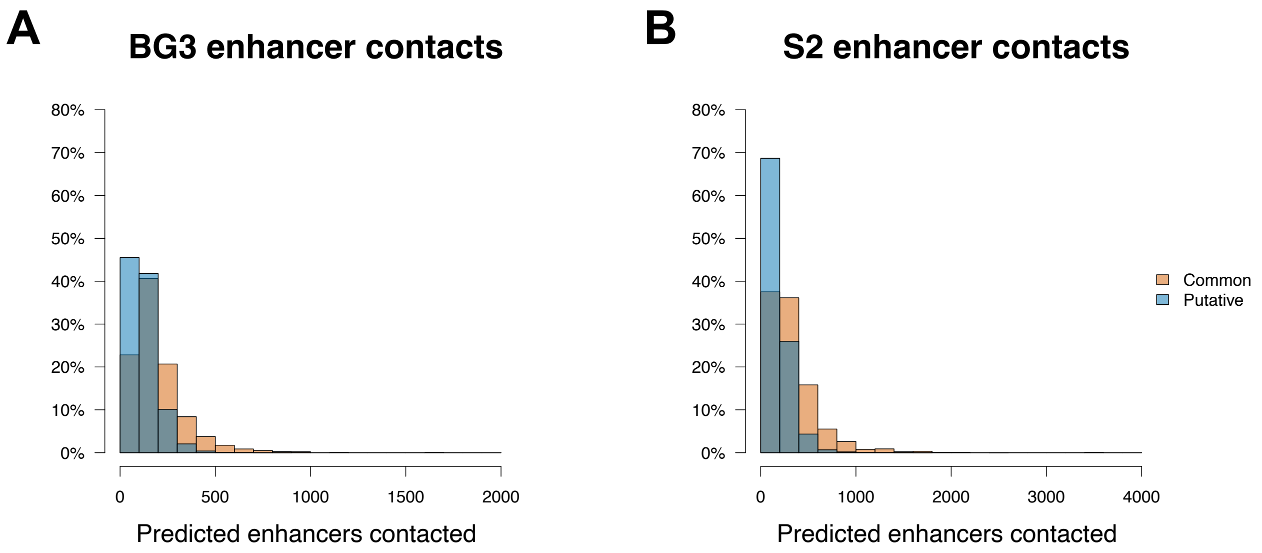

Fig. S6. *Co-localisation of enhancers in 3D.* Distributions of the number of Hi-C enriched contacts between enhancers. We considered the case of putative and common enhancers separately. Distributions shown as a percentage density of enhancers within a given bin (Mann-Whitney U test of distributions between common and putative enhancers; p-value $< 2.2\times10^{-16}$ for both BG3 and S2).

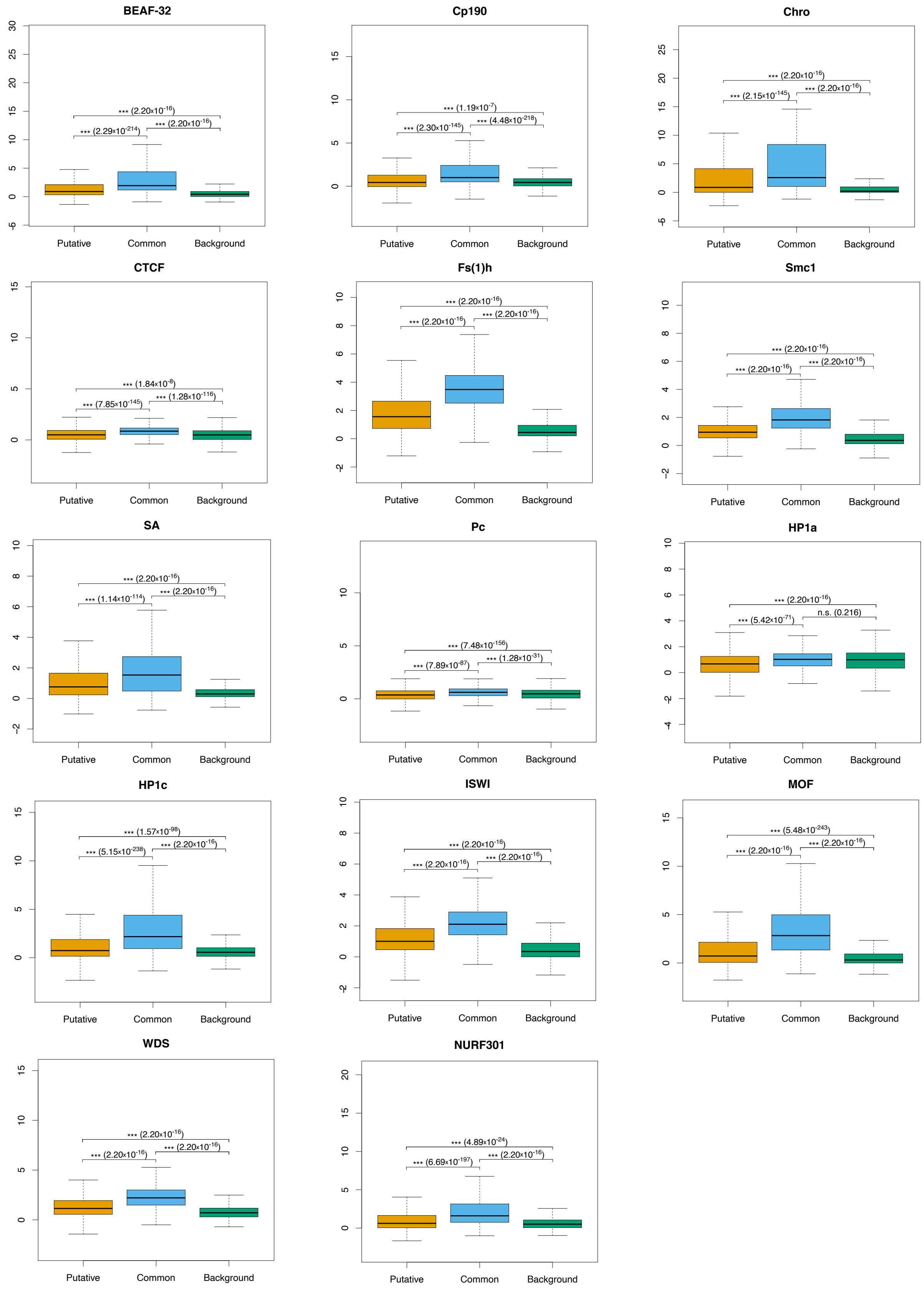

Fig. S7. *Different chromatin features and architectural proteins are enriched differently across enhancer groups and background.* The 95th percentile score across the body of each common and putative enhancer was plotted. Mann-Whitney U test scores can be found on each plot for each group comparison.

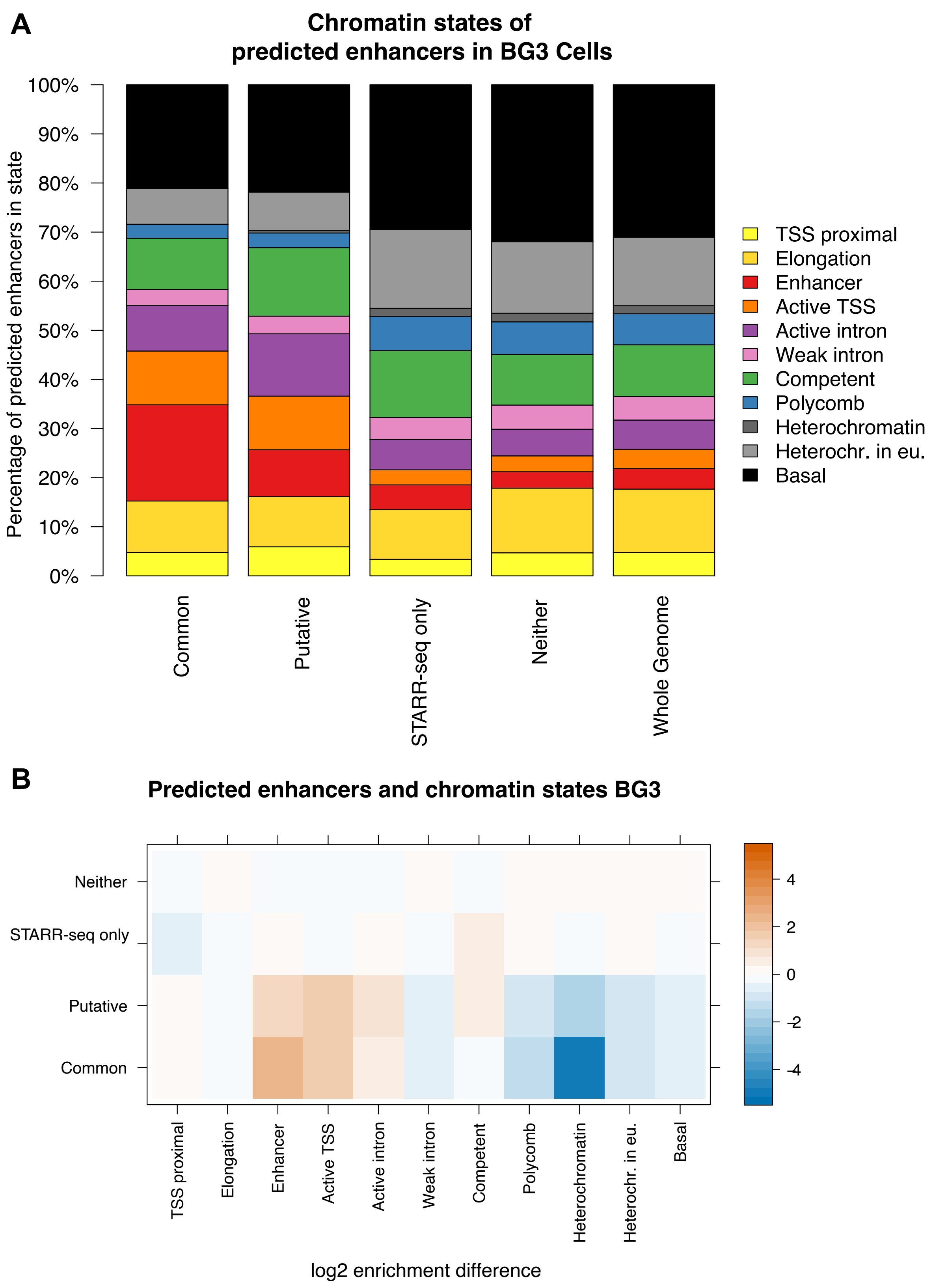

Fig. S8. *Overlap of BG3 enhancers with different chromatin states.* We used chromatin state annotation from [37]. (A) We considered different groups of enhancers: *(i)* enhancers detected by both STARR-seq and XAI (common enhancers), *(ii)* enhancers detected by XAI only (putative enhancers), *(iii)* enhancers detected by STARR-seq only and *(iv)* regions detected by neither. Overlap between the different groups of enhancers and 11 chromatin states. (B) $\log_2$(observed/expected) overlaps based on whole genome distribution of the different chromatin states.

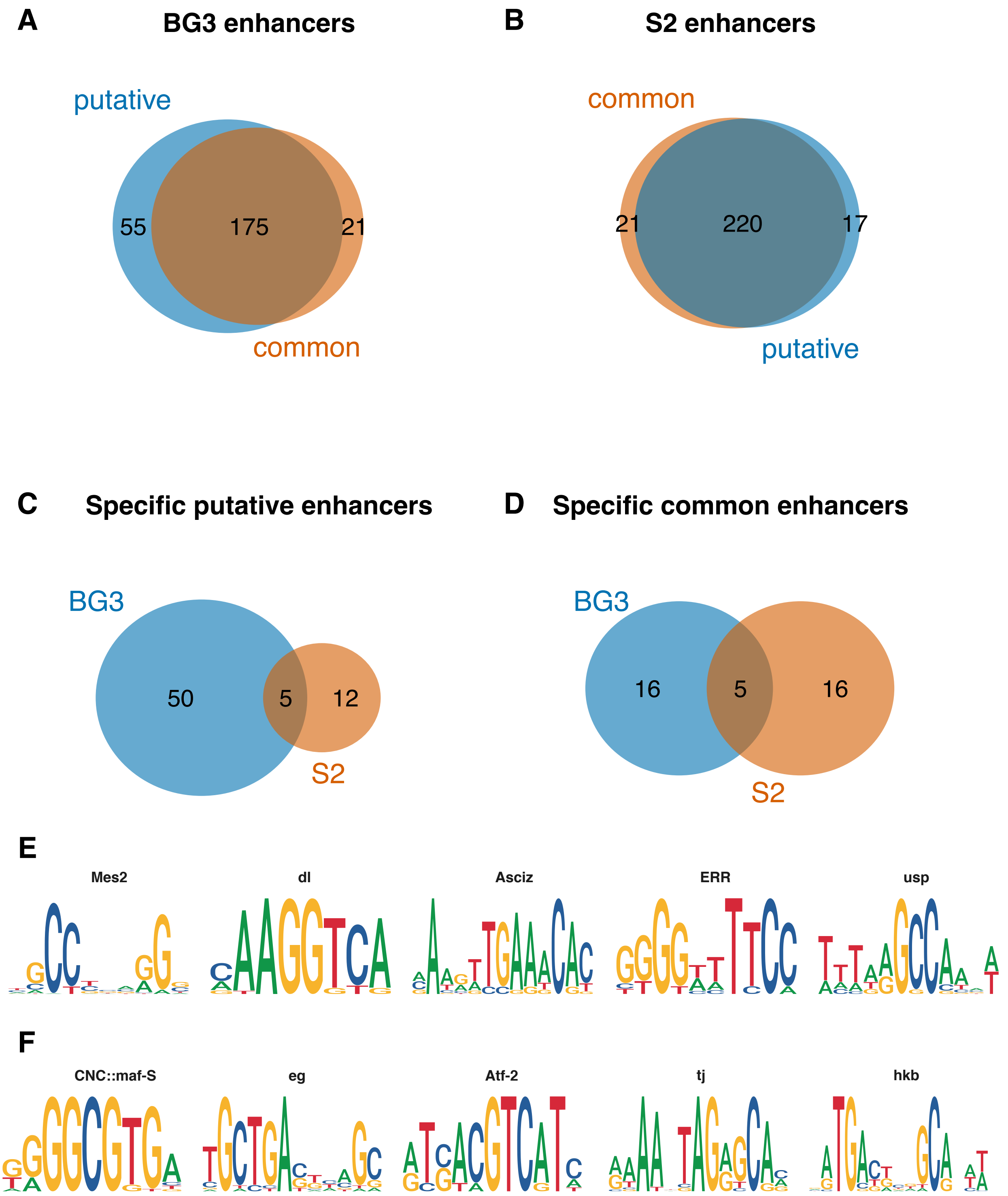

Fig. S9. *TF motif enrichment at common and putative enhancers.* We identified the enriched motifs in the common and putative enhancers (see Materials and Methods). (A-B) The overlap of enriched PWMs at common and putative enhancers in (A) BG3 cells and (B) S2 cells. (C) The overlap between specific motifs for putative enhancers in BG3 and in S2 cells. (D) The overlap between common specific motifs for enhancers in BG3 and in S2 cells. (E) The specific motifs for putative enhancers that are shared between BG3 and S2 cells. (F) The specific motifs for common enhancers that are shared between BG3 and S2 cells.

Supplementary Tables

Table S1: *Enriched motifs at common and putative enhancers in BG3 and S2 cells*

BG3 putative specific	S2 putative specific	BG3 common specific	S2 common specific
ems	ct	BtbVII	HLH54F
CG11617	ERR	brk	CG13897
NK7.1	dpn	Cf2_II	toy
Dfd	CG8281	amos	Kr
Awh	CG14962	Sox14	E(spl)mdelta-HLH
abd-A	Hr4	CNC::maf-S	l(1)sc
bap	Mes2	gt	eg
Antp	usp	eg	E(spl)m3-HLH
Scr	CG11504	jigr1	E(spl)mbeta-HLH
Ubx	dimm	Atf-2	sug
ind	bcd	gsb-n	CNC::maf-S
exex	Hr39	tj	Atf-2
otp	ftz-f1	STAT92E	Adf1
CG4136	Rel	Rel	NFAT
repo	dl_2	Mio	Su(H)
E5	KR	hkb	Usf
CG4404	Sox14	Optix	disco
CG11294		cwo	hkb
Lim3		ken	tj
Eip93F		CG6276	dys
Hnf4		vri	shn
lab			
btn			
CG15601			
PHDP			
C15			
Vsx1			
Mes2			
CG7056			
NFAT			
dl_2			
CG18599			
odd			
Atf6			
CG14962			
sens			
CG10904			
HGTX			
Eip75B			
Fer3			
Six4			
CG3065			

gl			
E(spl)m8-HLH			
pfk			
EcR::usp			
Hr83			
sens-2			
E(spl)			
CrebA			
disco			
CG32105			
ERR			
CG3919			
usp			

Table S2: *Datasets used in this study*

BG3 Histone Modifications			dm3 or dm6	LiftOver to dm6
H2Bubi	288	GSE20771	dm3	yes
H3K18ac	291	GSE20774	dm3	yes
H3K23ac	293	GSE20776	dm3	yes
H3K27ac	295	GSE20778	dm3	yes
H3K27me3	297	GSE20780	dm3	yes
H3K36me1	299	GSE20782	dm3	yes
H3K36me3	301	GSE20783	dm3	yes
H3K79me2	306	GSE20788	dm3	yes
H3K9me2	310	GSE20791	dm3	yes
H3K9me3	312	GSE20793	dm3	yes
H4K16ac	316	GSE20795	dm3	yes
H3K4me3	967	GSE20839	dm3	yes
H3K4me1	2653	GSE23468	dm3	yes
H3K4me2	2654	GSE23469	dm3	yes
H3K9acS10P	2659	GSE23474	dm3	yes
H3K27me2	2999	GSE27789	dm3	yes
H3K79me1	3005	GSE32736	dm3	yes
H4K20me1	3286	GSE32755	dm3	yes
H1	3299	GSE32767	dm3	yes
H3	3302	GSE32769	dm3	yes
H3K9ac	3765	GSE32832	dm3	yes
H3K9me1	3768	GSE32831	dm3	yes
H3K27me1	3941	GSE51965	dm3	yes
H3K79me3	4934	GSE45062	dm3	yes
H4K8ac	5060	GSE45070	dm3	yes
H2Av	6073	GSE45110	dm3	yes
**BG3 STARR-seq Datasets**				
STARR-seq peak summits	Yáñez-Cuna JO, *et al.*, 2014	GSE49809	dm3	yes
**S2 Histone Modifications**			**dm3 or dm6**	**LiftOver to dm6**
H2Bubi	290	GSE20773	dm3	yes
H3K18ac	292	GSE20775	dm3	yes
H3K23ac	294	GSE20777	dm3	yes
H3K27ac	296	GSE20779	dm3	yes
H3K27me3	298	GSE20781	dm3	yes
H3K36me3	303	GSE20785	dm3	yes
H3K4me1	304	GSE20786	dm3	yes
H3K79me2	307	GSE20789	dm3	yes
H3K9ac	309	GSE20790	dm3	yes
H3K9me3	313	GSE20794	dm3	yes
H4K16ac	319	GSE20798	dm3	yes
H4K8ac	322	GSE20801	dm3	yes

H3K4me2	2655	GSE23470	dm3	yes
H3K79me1	2658	GSE23473	dm3	yes
H3K9acS10P	2660	GSE23475	dm3	yes
H2Av	2991	GSE27731	dm3	yes
H3K27me2	3000	GSE27790	dm3	yes
H3K9me2	3011	GSE27741	dm3	yes
H4K20me1	3014	GSE27743	dm3	yes
H3K36me1	3170	GSE25374	dm3	yes
H1	3300	GSE32768	dm3	yes
H3	3301	GSE44465	dm3	yes
H3K4me3	3761	GSE32827	dm3	yes
H3K9me1	3770	GSE32833	dm3	yes
H3K27me1	3943	GSE44507	dm3	yes
H3K79me3	5143	GSE45090	dm3	yes
**S2 STARR-seq Datasets**				
STARR-seq peak summits	Yáñez-Cuna JO, *et al.*, 2014	GSE49809	dm3	yes
**S2 Developmental And Housekeeping Enhancers**				
STARR-seq peak summits	Zabidi, *et al.*, 2014	GSE57876	dm3	yes
**Architectural proteins**			**dm3 or dm6**	**LiftOver to dm6**
BEAF-32	3665	GSE32775	dm3	yes
Cp190	3666	GSE32776	dm3	yes
Criz/Chro	275	GSE20761	dm3	yes
CTCF	3673	GSE32783	dm3	yes
NippedB	Pherson et al (2019)	GSE118484	dm3	yes
**Transcription and replication**				
Pol-II	950	GSE20832	dm3	yes
3'NT-seq	Pherson et al (2017)	GSE100545	dm3	yes
**DNA accessibility**				
H3	3302	GSE32769	dm3	yes
H4	3303	GSE32770	dm3	yes
**Histone modifications (for Figure 6)**				
H3K4me3	967	GSE20839	dm3	yes
H3K4me1	2653	GSE23468	dm3	yes
H3K27ac	295	GSE20778	dm3	yes
H3K36me3	301	GSE20783	dm3	yes
H3K79me1	3005	GSE32736	dm3	yes
H4K16ac	316	GSE20795	dm3	yes
**Nucleosome remodelling factors**				
ISWI	3030	GSE27750	dm3	yes
MOF	3041	GSE27803	dm3	yes
WDS	5148	GSE45094	dm3	yes
NURF301	5063	GSE45072	dm3	yes

**Polycomb and heterochromatin**				
Pc	325	GSE20803	dm3	yes
HP1a	4126	GSE44515	dm3	yes
HP1c	942	GSE20824	dm3	yes
