# Supplementary material for: An explainable artificial intelligence approach for decoding the enhancer histone modifications code and identification of novel enhancers in Drosophila
Source: Genome Biol. 2021 Nov 8;22:308. doi: 10.1186/s13059-021-02532-7 (PMC8574042; doi:10.1186/s13059-021-02532-7)
Supplement: Supplementary file 2 — Additional file 2:. Review history [file 13059_2021_2532_MOESM2_ESM.docx]

Review History

**First round of review**

**Reviewer 1**

**Are you able to assess all statistics in the manuscript, including the appropriateness of statistical tests used?**

No, I do not feel adequately qualified to assess the statistics.

**Comments to author:**

This manuscript reports the use of machine learning and rule based explainable AI models to predict enhancers in the Drosophila genome. The authors used as training datasets input data corresponding to various histone modifications generated by the modENCODE project in Drosophila cell lines, and experimentally validated enhancers from STARR-seq experiments. The prediction accuracy from machine learning and rule based AI appears to be quite good, and importantly reveled certain correlations of histone modifications, chromatin binding proteins at different classes of enhancers. The machine learning and XAI approaches are powerful and methodologies will be of high interest to the field of regulatory genomics.

Minor comments -

1. Please explain "housekeeping" enhancers.

2. Please add more description on the different rules of enhancers.

3. Please explain the differences between XAI rules learning from BG3 and S2 cells - shouldn't they be similar?

4. It would be nice to test the novel enhancer predictions that were not tested or tested negative by STARR-seq.

**Reviewer 2**

**Are you able to assess all statistics in the manuscript, including the appropriateness of statistical tests used?**

Yes, and I have assessed the statistics in my report.

**Comments to author:**

Major concerns: There is little support in the manuscript for calling the predicted enhancers a "novel" set of enhancers. According to this report, the putative enhancers feature very similar characteristics to common enhancers and are numerous, which makes them more likely to be either redundant enhancers or not enhancers at all. Moreover, the authors didn't provide sufficient evidence to support the active state and novelty of discovery of the predicted enhancers. They didn't describe how these predicted enhancers differ from the common enhancers in sufficient detail. More importantly, the contribution of these putative enhancers to cellular processes in Drosophila was not discussed. I didn't find sufficient evidence, which would conclusively prove the existence of a new class of enhancers in Drosophila, which has not been identified in the past. In particular, what are the genes regulated by this novel set of enhancers, what is the function of these genes, and what is the role of the novel enhancers in the global regulatory program. Do they regulate target genes independently or in a cooperative way? Do they interact with the "classical" enhancers? Could they be paused enhancers? Experimental validation of enhancer activity of at least a handful of these "novel" enhancers is needed.

Minor: I understand the motivation of the authors to predict enhancers in the S2 cell line using the model trained on BG3 cells. What is the performance in predicting S2 enhancers using the S2 model, as well as the performance in predicting BG3 enhancers using the S2 model?

Figure 2C: There is a peak at 40 bp for putative enhancers, would the authors comment on that?

Figure 3A: The authors used genome-wide Hi-C data. Why did the author split enhancers into proximal (<5kb) and distal, and only calculate Hi-C contacts for distal enhancers? Why not calculate Hi-C contacts for all enhancers, since enhancers might contact distal promoters by skipping several nearby genes?

Figure 3D: I don't see a big difference in trends between putative enhancers and the whole-genome background. I'd recommend using a more suitable background for this test.

Figure 5D: The rules 42 and 37 shows "high level of H3K4me1 and H3K27ac define enhancer", compared to no such rules in the whole set of enhancers (Figure 4C). Would the authors comment on that?

Figure 6: The subplots referring to NT-seq, bidirectional, and TAD borders have not been discussed in the main text.

Page 4, lines 48-49: Please list the exact numbers after the "increased number of 3D contacts".

Page 4, line 9: Should the "STARR-seq" be changed to "common"?

Page 5, line 38: "Figure 4C-F" should be "Figure 4C-E".

Page 5, line 55: "Figure S5D-E" should be "Figure 5D-E".

**Authors Response**

**Point-by-point responses to the reviewers’ comments:**

Reviewer #1

This manuscript reports the use of machine learning and rule based explainable AI models to predict enhancers in the Drosophila genome. The authors used as training datasets input data corresponding to various histone modifications generated by the modENCODE project in Drosophila cell lines, and experimentally validated enhancers from STARR-seq experiments. The prediction accuracy from machine learning and rule based AI appears to be quite good, and importantly revealed certain correlations of histone modifications, chromatin binding proteins at different classes of enhancers. The machine learning and XAI approaches are powerful and methodologies will be of high interest to the field of regulatory genomics.

*Thank you for your comments, we appreciate your feedback and feel that the suggestions below will improve the clarity of our manuscript.*

Minor comments -

1. Please explain "housekeeping" enhancers.

*We have added a sentence in the text explaining the difference between housekeeping and developmental enhancers.*

*“Housekeeping enhancers are enhancers expressed across multiple cell types and are crucial to the correct functioning of the cell, while developmental enhancers are enhancers that are cell type specific and are involved in differential gene expression across various cell types.”*

2. Please add more description on the different rules of enhancers.

*We have added additional explanations and examples in the main text.*

*“Individual classifications of epigenetic marks as low, medium and high do not have hard borders but instead allow for some overlap between classes. These boundaries vary by mark and are trained when training the model. Interestingly, the rules for enhancers contain high levels of H3K4me1 together with either high level of H4K16ac or H3K18ac, but not with high levels of H3K27ac (Figure 4A). Nevertheless, 70% of the regions displaying high levels of both H3K4me1 and H3K27ac are selected by our explainable AI model and, consequently, can be explained by other combinations of histone modifications. Interestingly, it was recently shown that H3K27ac is not required for enhancer activity and its depletion at enhancers in mouse ES cells results in only a few small changes in gene expression [18].*

*…*

*We found that approximately 9% of all predicted enhancers in BG3 cells are characterised by high levels of H3K4me1 and H4K16ac. One example is rule 47 which triggers when H3K4me1 enrichment is high, H3K16ac is high and H2K23ac is low (Figure 4A).*

*Figure 4B contains rules used to classify regions as non-enhancers and not surprisingly non-enhancers are depleted in H3K4me1. For example, rule 19 states that if H3K18ac and H3K4me1 are low, then the region will be classified as a non-enhancer. Non enhancers are also characterised by depletion of H3K4me2 and H3K9me2 or enrichment of H3, H3K27me3 and H3K4me3.*

*Interestingly, enrichment of H3K4me3 is usually associated with promoters [12], but broad H3K4me3 peaks have been previously associated with enhancers [47]. Our results show that when high levels of H3K4me3 are associated together with depletion of H3K27me3 or depletion of H2AV, then those regions are classified as enhancers (Figure 4A). Furthermore, high levels of H3K4me3 together with medium levels of H3K36me1 or H3K18ac characterised regions that are not classified as enhancers (Figure 4B). ”*

3. Please explain the differences between XAI rules learning from BG3 and S2 cells - shouldn't they be similar?

*In this manuscript, we only generated the set rules in BG3 cells because we aimed to show that the model generalises well, despite it having not been previously trained on S2 data. Nevertheless, the fact that the rules for a BG3 trained model perform similarly well in S2 cells, indicates that our rules are general across different cell types, being independent on the cell the model is trained on. We have added a sentence in the introduction to clarify this.*

*“Our main aim is to evaluate how successfully the model generalises to a different cell line that it was never trained on and, thus, we only trained the model in BG3 cells. Nevertheless, the performance of the model in S2 cells, indicates, that the BG3 rules are generalisable and can explain enhancers in other cell types.”*

4. It would be nice to test the novel enhancer predictions that were not tested or tested negative by STARR-seq.

*We agree with this point and have added an additional analysis to show that these putative enhancers display intermediary levels of nascent RNA. While these levels are lower compared to the values observed at STARR-seq enhancers, they are above the genomic background and these differences are statistically significant. This indicates that the putative enhancers have a capacity to induce transcription, albeit lower compared to STARR-seq enhancers. We have added the following explanation in the main text. Also note that following suggestions from reviewer 2, we performed additional analysis which discovered some differences between putative and common enhancers.*

*“Enhancers are often characterised by active transcription, usually divergent transcription [58–61]. We observed an enrichment of 3’NT-seq signal across both common and putative enhancers (Figure 6A), suggesting that both groups of enhancers are transcriptionally active. Furthermore, both common and putative enhancers display strong bidirectional transcription (Figure 6A), a characteristic of enhancers [58,59]. What differentiates putative and common enhancers, is the level of nascent RNA displayed, with putative enhancers having an intermediary level of nascent RNA, while common enhancers have the highest level of nascent RNA (Figure 6E). This result is further mirrored by Pol-II signal, which is higher at common enhancers than at putative ones (Figure 6E). Altogether, our results show that while putative enhancers display activity, this is lower than the activity of common enhancers, which can potentially explain why they were not detected with the original STARR-seq plasmid.”*

Reviewer #2:

Major concerns:

1. There is little support in the manuscript for calling the predicted enhancers a "novel" set of enhancers. According to this report, the putative enhancers feature very similar characteristics to common enhancers and are numerous, which makes them more likely to be either redundant enhancers or not enhancers at all. Moreover, the authors didn't provide sufficient evidence to support the active state and novelty of discovery of the predicted enhancers. They didn't describe how these predicted enhancers differ from the common enhancers in sufficient detail.

More importantly, the contribution of these putative enhancers to cellular processes in Drosophila was not discussed. I didn't find sufficient evidence, which would conclusively prove the existence of a new class of enhancers in Drosophila, which has not been identified in the past. In particular, what are the genes regulated by this novel set of enhancers, what is the function of these genes, and what is the role of the novel enhancers in the global regulatory program. Do they regulate target genes independently or in a cooperative way? Do they interact with the "classical" enhancers? Could they be paused enhancers? Experimental validation of enhancer activity of at least a handful of these "novel" enhancers is needed.

*We would want to thank the reviewer for their helpful comments that improved our manuscript.*

*Firstly, we should clarify that we were not implying that we have identified a novel class of enhancers in Drosophila, but that we identified potential enhancers that were not previously detected by STARR-seq. Some of these enhancers that were not identified from STARR-seq overlapped those in the Enhancer Atlas (highlighted in figure S3) but not all of them. Furthermore, it was recently shown that the original STARR-seq plasmid potentially missed enhancers and a new plasmid was introduced (see Muerdeter et al., 2017 https://www.nature.com/articles/nmeth.4534). Our putative enhancers were only “novel” as they were not yet identified by the original STARR-seq assay. We clarified this in the text as follows.*

*“We know that STARR-seq recovered enhancers are dependent on the plasmid used in the experiment, suggesting that it could lead to an increase in false negatives (true enhancers that are missed) [24]. In particular, the STARR-seq datasets we used in this study were generated using an older version of the plasmid that has been shown to miss enhancers [24]. In addition, some of these putative enhancers (approximately 2,500), albeit not all of them, have been previously characterised as enhancers by other methods in Enhancer Atlas 2.0 [26] (Figure S3).”*

*Second, reviewer 2 suggestion that some of the enhancers could be redundant is a valid point. We performed an additional analysis to evaluate how many enhancers were in 3D proximity to other enhancers. Figure S6 shows that both putative and common enhancers display enriched contacts with other enhancers, but common enhancers have a higher number of contacts with other enhancers than putative ones. We addressed this in the main text as follows:*

*“There also exists the possibility that these putative enhancers are redundant enhancers. To investigate this, we plotted the enriched Hi-C contacts of putative and common enhancers to all other predicted enhancers (including both putative and common) (Figure S6). Common enhancers tended to contact more enhancers, but whether this is due to the common enhancers themselves generally being larger or another mechanism is not clear.*

*…*

*Enhancer hubs have been previously observed in many organisms and there is evidence that these redundant enhancers have a role in providing robustness in gene activation [45,46]. Here we found that putative enhancers display high number of enriched contacts with other enhancers, albeit less than in the case of common enhancers (Figure S6). One possibility is that our putative enhancers are potentially redundant enhancers that are part of enhancer hubs.”*

*Third, the reviewer suggested to evaluate the activity state of the enhancers. We know that enhancers produce nascent transcript and also display divergent transcription (Bose et al., 2017 https://www.cell.com/cell/fulltext/S0092-8674(16)31736-6; Henriques et al., 2018 http://genesdev.cshlp.org/content/32/1/26). Thus, we measured the levels of nascent RNA at putative and common enhancers and divergent or bidirectional transcription. Our results confirm that both putative and common enhancers display strong bidirectionality and levels of nascent RNAs above background. Nevertheless, the putative enhancers showed lower levels of nascent RNAs compared to common enhancers suggesting that they have lower activity. This may also explain why they were not detected with the original STARR-seq plasmid.*

*“Enhancers are often characterised by active transcription, usually divergent transcription [58–61]. We observed an enrichment of 3’NT-seq signal across both common and putative enhancers (Figure 6A), suggesting that both groups of enhancers are transcriptionally active. Furthermore, both common and putative enhancers display strong bidirectional transcription (Figure 6A), a characteristic of enhancers [58,59]. What differentiates putative and common enhancers, is the level of nascent RNA displayed, with putative enhancers having an intermediary level of nascent RNA, while common enhancers have the highest level of nascent RNA (Figure 6E). This result is further mirrored by Pol-II signal, which is higher at common enhancers than at putative ones (Figure 6E). Altogether, our results show that while putative enhancers display activity, this is lower than the activity of common enhancers, which can potentially explain why they were not detected with the original STARR-seq plasmid.”*

*Fourth, the reviewer asked if there is any difference between putative and common enhancers that could explain why the former are not detected by STARR-seq. To address this, we trained an explainable AI model to classify putative or common enhancers. This model performed with a high degree of accuracy (avg. recall 77.3% across the whole dataset and AUC of 0.83); Figure 6B. The highest dominance rules generated by this model can be found in the revised Figure 6C. We found two primary features to be of the most important in differentiating putative and common enhancers, namely: Mediator complex and Cohesin. To avoid splitting the rules, we used in our model a single subunit for Mediator complex (Med1) and one for cohesin (Nipped-B). Mediator and cohesin are key complexes enriched at enhancers. While these elements are highly enriched at common enhancers, they show medium enrichment at putative enhancers that is still significantly above the background level, as shown in Figures 6E and S6. To better observe these differences between putative and common enhancers, we used a different colour scheme for the heatmaps, which improves clarity.*

*“To further evaluate potential subtle differences between the common and putative enhancers, we trained an additional XAI model to predict whether a given enhancer is common or putative based on features in Figure 6A. The model we trained was able to predict which group an enhancer belonged to with a 77.3% average recall and 0.83 AUC using a set of 16 features (Figure 6B-C). Due to the similarities in function of subunits of cohesin and the mediator complex we used a single track for each of the two complexes (Nipped-B for cohesin and MED1 for Mediator) to prevent rules involving cohesin or the mediator complex from becoming diluted across several features. In particular, Nipped-B and SA recruit cohesin to the genome and Rad21 and Smc1 are two cohesion subunits [62]. Fs(1)h was found to recruit Nipped-B [63] and, thus, was also not included in the selected features. Mediator (MED1) appeared in 9 of the 10 and cohesin (Nipped-B) in 6 out of 10 high dominance rules predicting common enhancers. These features show a similar distribution between common and putative enhancers, but putative enhancers have a reduced intensity across these tracks (Figure 6A). Furthermore, we plotted the distributions of all of these features at common, putative and background regions (Figures 6E and S7). Our results show that all of the cohesin and mediator subunits display higher enrichment at common enhancers than at putative ones, but these features are still more enriched in putative enhancers than they are across the genomic background. We hypothesise that the reduced recruitment of cohesin and mediator complex at these enhancers may prevent them to display similarly high activity as common enhancers. Furthermore, other architectural proteins (BEAF-32, Cp190, Chro and CTCF) or chromatin remodellers (ISWI, MOF, WDS and NURF301) show similar strong enrichment at common enhancers and medium enrichment at putative enhancers (Figure S7). ”*

*“Our results show medium enrichment of cohesin (Rad21, Smc1, Nipped-B and SA) and architectural proteins (BEAF-32, Cp190, Chro and CTCF) [35,77] at putative enhancers. This is in contrast with the higher enrichment of these proteins at common enhancers (Figure 6 and S7). The lower enrichment of cohesin at putative enhancers correlates with lower activity (nascent RNA) that can lead to the enhancer displaying an activity below the detection threshold of high throughput enhancer assays (such as STARR-seq or other massively parallel reporter assays). Interestingly, recent studies have shown that cohesin is required for enhancer activity in mammalian systems, but mainly for long range enhancer activity [78,79]. We find that the 3D genome architecture might play a role in enhancer activity in Drosophila similarly to mammalian system [62]. ”*

Minor:

2. I understand the motivation of the authors to predict enhancers in the S2 cell line using the model trained on BG3 cells. What is the performance in predicting S2 enhancers using the S2 model, as well as the performance in predicting BG3 enhancers using the S2 model?

*We have performed the suggested analysis by the reviewer and this led to the model overfitting. In particular, we trained the model on S2 cells and the performance was similar as in the case of the BG3 trained model, see new Figure S1A (Accuracy and Average Recall between 0.75-0.80). Nevertheless, the model trained on S2 cells did not generalise well and when it was applied on data in BG3 cells and we obtained a low performance (Accuracy and Average Recall between 0.35-0.60). This indicates that the S2 trained model is overfitted to S2 cell data and cannot generalise well. Thus, we did not proceed for the downstream analysis with this model. We added in the main text the following text:*

*“To investigate how well the ML and explainable AI models generalise, we trained the models on data from BG3 cells and predicted enhancers in S2 cells using the corresponding histone modifications ChIP datasets (Figure 1B). Although the models had not previously been exposed to data from S2 cells, it performed with a similar degree of accuracy, highlighting its ability to generalise to new cell lines in different tissues and developmental stages. Furthermore, we also trained a model on S2 cell data, but, while this model performed well in S2 cells, it had a very low accuracy in BG3 cells (Figure S1). This indicates that the S2 trained model did not generalise and, thus, for the downstream analysis we used only the BG3 trained model.”*

3. Figure 2C: There is a peak at 40 bp for putative enhancers, would the authors comment on that?

*We believe these are artefacts of noise being predicted in small, localised bins. We have amended the text to highlight that.*

*“As the model predicts individual 10 bp bins, noisy regions in ChIP signals can lead to small false positive regions being predicted as enhancers. Thus, enhancers shorter than 50 bp are likely to be artefacts and in the downstream analysis we only consider enhancers larger than 50 bp.”*

4. Figure 3A: The authors used genome-wide Hi-C data. Why did the author split enhancers into proximal (<5kb) and distal, and only calculate Hi-C contacts for distal enhancers? Why not calculate Hi-C contacts for all enhancers, since enhancers might contact distal promoters by skipping several nearby genes?

*Thank you for pointing out that this was not clear in the figure legend. Where enhancers are listed as proximal, they sit within 5 kb of a promoter, but also have distal contacts with promoters when we calculated their Hi-C contacts. Where enhancers are listed as “Proximal Only”, they sit within 5 Kb of a promoter, but we observed no Hi-C contacts with distal promoters. We have updated the figure legend to clarify this point.*

*“Figure 3. Putative enhancers make 3D contacts with expressed genes. (A) We split enhancers into: (i) proximal if they are located within 5 Kb of a promoter, (ii) distal if they are further than 5Kb from any promoters and make 3D contact with promoters, (iii) proximal only if they do not have enriched Hi-C contacts more than 5kb away but were within 5 Kb of a promoter and (iv) neither if they are further than 5Kb from any promoter and do not make 3D contacts with any promoters.”*

5. Figure 3D: I don't see a big difference in trends between putative enhancers and the whole-genome background. I'd recommend using a more suitable background for this test.

*The reviewer is correct that the distributions look similar. Nevertheless, when we compared the means of the expression of genes contacted by putative enhancers and background regions, we can observe a difference that is statistically significant. We updated the Figure legend to include this.*

*“In BG3 cells, distal only enhancers have a mean log2 of FPKM of 9.76, while distal background regions of 9.6 (p-value 2.1×10-40). Similarly, in BG3 cells, proximal enhancers have a mean log2 of FPKM of 10.40 and proximal background regions of 9.88 (p-value 1.3×10-100). In S2 cells, distal only enhancers have a mean log2 of FPKM of 7.97, while distal background regions of 7.70 (p-value 1.0×10-31). Similarly, in S2 cells, proximal enhancers have a mean log2 of FPKM of 8.17 and proximal background regions of 7.75 (p-value 3.6×10-58). Note that in each case we performed a Mann Whitney U Test.”*

6. Figure 5D: The rules 42 and 37 shows "high level of H3K4me1 and H3K27ac define enhancer", compared to no such rules in the whole set of enhancers (Figure 4C). Would the authors comment on that?

*This is a very interesting point raised by the reviewer. Figure 5 presents the rules used to distinguish between developmental and housekeeping enhancers. High levels of H3K4me1 and H3K27ac is the top rule to identify developmental enhancers from the list of total enhancers (housekeeping and developmental). Nevertheless, the model performs poorly at differentiating between developmental enhancers and housekeeping ones (see Figure 5B and C). In Figure 4C, the rules are used to identify enhancers (including both housekeeping and developmental) from the entire genome. Interestingly, we do add this rule (High H3K4me1 and H3K27ac) to our existing model in Figure 4C (Rule 1), but this improves the model’s average recall by slightly less than 0.5% (Figure 4E).*

*“Interestingly, the rule “high levels of H3K4me1 and H3K27ac” was able to only negligibly improve the model performance for our enhancer annotation (Figure 4C), but is associated with developmental enhancers and not housekeeping enhancers (Figure 5D). Altogether, our results support a model where there are marginal epigenetic differences between housekeeping and developmental enhancers, but these differences were not sufficient to properly distinguish between the two.”*

7. Figure 6: The subplots referring to NT-seq, bidirectional, and TAD borders have not been discussed in the main text.

*In the revised manuscript, all these subplots have been discussed. For the 3’NT-seq and bidirectional transcription, see response to major concern (third paragraph). For TADs, we added the following explanation.*

*“Previously, we found that BG3 specific TAD borders display enhancer like epigenetic landscape, but were not identified as enhancers by STARR-seq [35]. Furthermore, the enrichment of architectural proteins and common and putative enhancers raised the question whether these enhancers are associated with TAD borders or not. Nevertheless, we found that only a small number of common and putative enhancers are located within 2 Kb of TAD borders (Figure 6A). ”*

8. Page 4, lines 48-49: Please list the exact numbers after the "increased number of 3D contacts".

*That was added in the revised version of the manuscript.*

*“The increased high number of 3D contacts between the genomic background and promoters (4.7M in BG3 and 5.9M in S2) can be explained by the fact that the genomic background contains many exons and introns (see Figure 2B) and expressed genes in Drosophila form gene domains with many enriched 3D contacts [44].”*

9. Page 4, line 9: Should the "STARR-seq" be changed to "common"?

*This has been corrected in the text.*

10. Page 5, line 38: "Figure 4C-F" should be "Figure 4C-E".

*This has been corrected in the text, thank you for spotting it.*

11. Page 5, line 55: "Figure S5D-E" should be "Figure 5D-E".

*This has been corrected in the text, thank you for spotting it.*

**Second round of review**

**Reviewer 1**

The authors have adequately addressed my previous comments. The revised manuscript is a very timely contribution to the study of enhancer biology. The XAI and ML modeling approaches described here are likely to have general utilities, while the enhancer rules would provide new hypotheses for future studies. I recommend acceptance.
